# In-depth phosphoproteomic profiling of the insulin signaling response in heart tissue and cardiomyocytes unveils canonical and specialized regulation

**DOI:** 10.1186/s12933-024-02338-4

**Published:** 2024-07-18

**Authors:** Jonathan Samuel Achter, Estefania Torres Vega, Andrea Sorrentino, Konstantin Kahnert, Katrine Douglas Galsgaard, Pablo Hernandez-Varas, Michael Wierer, Jens Juul Holst, Jørgen Frank Pind Wojtaszewski, Robert William Mills, Rasmus Kjøbsted, Alicia Lundby

**Affiliations:** 1 Department of Biomedical Sciences, Faculty of Health and Medical Sciences, University of Copenhagen, Copenhagen, Denmark; 2 Core Facility for Integrated Microscopy, Department of Biomedical Sciences, Faculty of Health and Medical Sciences, University of Copenhagen, Copenhagen, Denmark; 3 Proteomics Research Infrastructure, Faculty of Health and Medical Sciences, University of Copenhagen, Copenhagen, Denmark; 4 The Novo Nordisk Foundation Center for Basic Metabolic Research, Faculty of Health and Medical Sciences, University of Copenhagen, Copenhagen, Denmark; 5 The August Krogh Section for Molecular Physiology, Department of Nutrition, Exercise and Sports, Faculty of Science, University of Copenhagen, Copenhagen, Denmark

**Keywords:** Insulin signaling, Phosphoproteomics, Proteomics, Phosphorylation, Metabolism, Cardiometabolic, Insulin resistance, Cardiac signaling, Tbc1d4, Kinase

## Abstract

**Background:**

Insulin signaling regulates cardiac substrate utilization and is implicated in physiological adaptations of the heart. Alterations in the signaling response within the heart are believed to contribute to pathological conditions such as type-2 diabetes and heart failure. While extensively investigated in several metabolic organs using phosphoproteomic strategies, the signaling response elicited in cardiac tissue in general, and specifically in the specialized cardiomyocytes, has not yet been investigated to the same extent.

**Methods:**

Insulin or vehicle was administered to male C57BL6/JRj mice via intravenous injection into the vena cava. Ventricular tissue was extracted and subjected to quantitative phosphoproteomics analysis to evaluate the insulin signaling response. To delineate the cardiomyocyte-specific response and investigate the role of Tbc1d4 in insulin signal transduction, cardiomyocytes from the hearts of cardiac and skeletal muscle-specific Tbc1d4 knockout mice, as well as from wildtype littermates, were studied. The phosphoproteomic studies involved isobaric peptide labeling with Tandem Mass Tags (TMT), enrichment for phosphorylated peptides, fractionation via micro-flow reversed-phase liquid chromatography, and high-resolution mass spectrometry measurements.

**Results:**

We quantified 10,399 phosphorylated peptides from ventricular tissue and 12,739 from isolated cardiomyocytes, localizing to 3,232 and 3,128 unique proteins, respectively. In cardiac tissue, we identified 84 insulin-regulated phosphorylation events, including sites on the Insulin Receptor (Insr^Y1351, Y1175, Y1179, Y1180^) itself as well as the Insulin receptor substrate protein 1 (Irs1^S522, S526^). Predicted kinases with increased activity in response to insulin stimulation included Rps6kb1, Akt1 and Mtor. Tbc1d4 emerged as a major phosphorylation target in cardiomyocytes. Despite limited impact on the global phosphorylation landscape, Tbc1d4 deficiency in cardiomyocytes attenuated insulin-induced Glut4 translocation and induced protein remodeling. We observed 15 proteins significantly regulated upon knockout of *Tbc1d4*. While Glut4 exhibited decreased protein abundance consequent to Tbc1d4-deficiency, Txnip levels were notably increased. Stimulation of wildtype cardiomyocytes with insulin led to the regulation of 262 significant phosphorylation events, predicted to be regulated by kinases such as Akt1, Mtor, Akt2, and Insr. In cardiomyocytes, the canonical insulin signaling response is elicited in addition to regulation on specialized cardiomyocyte proteins, such as Kcnj11^Y12^ and Dsp^S2597^. Details of all phosphorylation sites are provided.

**Conclusion:**

We present a first global outline of the insulin-induced phosphorylation signaling response in heart tissue and in isolated adult cardiomyocytes, detailing the specific residues with changed phosphorylation abundances. Our study marks an important step towards understanding the role of insulin signaling in cardiac diseases linked to insulin resistance.

**Graphical Abstract:**

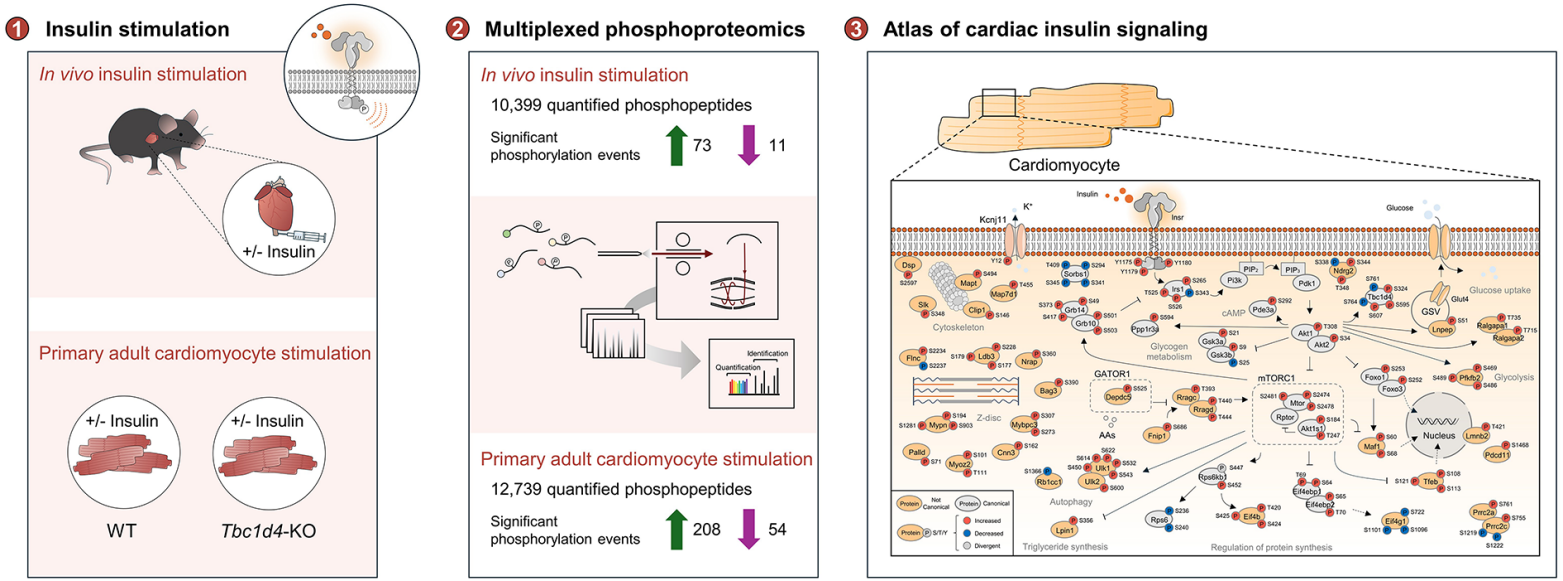

**Supplementary Information:**

The online version contains supplementary material available at 10.1186/s12933-024-02338-4.

## Introduction

Insulin signaling, with its diverse effects across metabolic organs [1], plays a significant role in the heart, where insulin receptors are highly expressed [2]. It is involved in various aspects of cardiac function and metabolism, including the regulation of cardiomyocyte growth, apoptosis, glucose and fatty acid uptake and metabolism [2–4]. Knock out of insulin receptors in cardiomyocytes highlighted the importance of insulin signaling for cardiac size and regulation of mitochondrial oxidative capacity [3, 5, 6]. The heart has the ability to tune the use of different substrates based on its physiological requirements and energy demand [7]. Cardiac metabolism is largely determined by substrate availability, with the heart primarily relying on fatty acids as its metabolic substrate [8]. Changes in circulating insulin affect myocardial metabolism by regulating myocardial substrate supply and by direct signaling effects on the myocardium. In the heart, there is an insulin-induced adaptation in substrate utilization. Under physiological conditions, the main substrate is mitochondrial oxidation of fatty acids, and to a lesser extent, glucose, as fatty acid oxidation inhibits glucose uptake and catabolism via the Randle cycle [9, 10]. Following a meal, when blood glucose and insulin levels rise, insulin regulation promotes glucose uptake and utilization via glycolysis [10]. However, under pathological conditions, such as type-2 diabetes or heart failure, cardiac insulin signaling is altered [2, 7, 10–12]. Impairments in insulin signaling among obese or diabetic patients are thought to contribute to their increased risk of heart failure [2]. Despite insulin signaling modulating critical processes within cardiomyocytes, details of the signaling response in the heart remain uncharacterized.

Protein phosphorylation is central to the insulin response, serving as a reversible post-translational modification that mediates insulin signal transduction. The process and details of protein phosphorylation are crucial for understanding insulin-modulated processes in the heart. The binding of insulin to the extracellular part of the insulin receptor activates the receptor’s intrinsic tyrosine kinase activity, leading to autophosphorylation of several tyrosine residues. This creates binding sites for signaling adapter proteins [13]. Adapter proteins, such as Insulin receptor substrate proteins 1 and 2 (Irs1 and Irs2) and SHC-transforming protein 1 (Shc1), bind to the receptor and become phosphorylated, triggering downstream signaling events via phosphoinositide-3-kinase, protein kinase B (Akt), or members of the Mitogen-activated protein kinase (Mapk) signaling pathway [2].

Significant progress has been made in understanding the signal transduction elements involved in insulin signaling in skeletal muscle, liver, adipocytes, and pancreatic beta cells. Mass spectrometry-based phosphoproteomics studies have revealed extensive phosphorylation networks activated by insulin stimulation in these tissues [14–21]. This methodology has also been effective in quantifying protein phosphorylation events in cardiac tissue [22, 23]. In this study, we employ quantitative phosphoproteomics to map the insulin signaling network in cardiac tissue and cardiomyocytes. Our objectives are to: *(1) decipher the global insulin signaling response in the heart in vivo, and (2) decipher the cell-specific protein phosphorylation response to insulin in isolated adult cardiomyocytes.* We found that TBC1 domain family member 4 (Tbc1d4) is a highly regulated protein in the heart upon insulin stimulation and with a particular expression profile in cardiomyocytes. Accordingly, we extended our investigation to also *(3) investigate the role of Tbc1d4 in insulin signal transduction in adult cardiomyocytes.* In the present work, we investigated the signaling response in cardiac tissue procured from mice after administration of either insulin or vehicle via the vena cava. This methodology was employed to prioritize the delivery of insulin to the target organ, thereby enabling a focused evaluation of the cardiac signaling response triggered by insulin. Investigating the extensive phosphorylation signaling network orchestrated by insulin in the heart and cardiomyocytes is a first step towards understanding insulin signal transduction that may be unique to the heart and aid in understanding how altered insulin signaling may contribute to pathophysiological remodeling in the heart in the setting of diabetes or heart failure.

## Results

### Inducing an insulin signaling response in cardiac tissue in vivo for phosphoproteomics evaluation

**Fig. 1:**
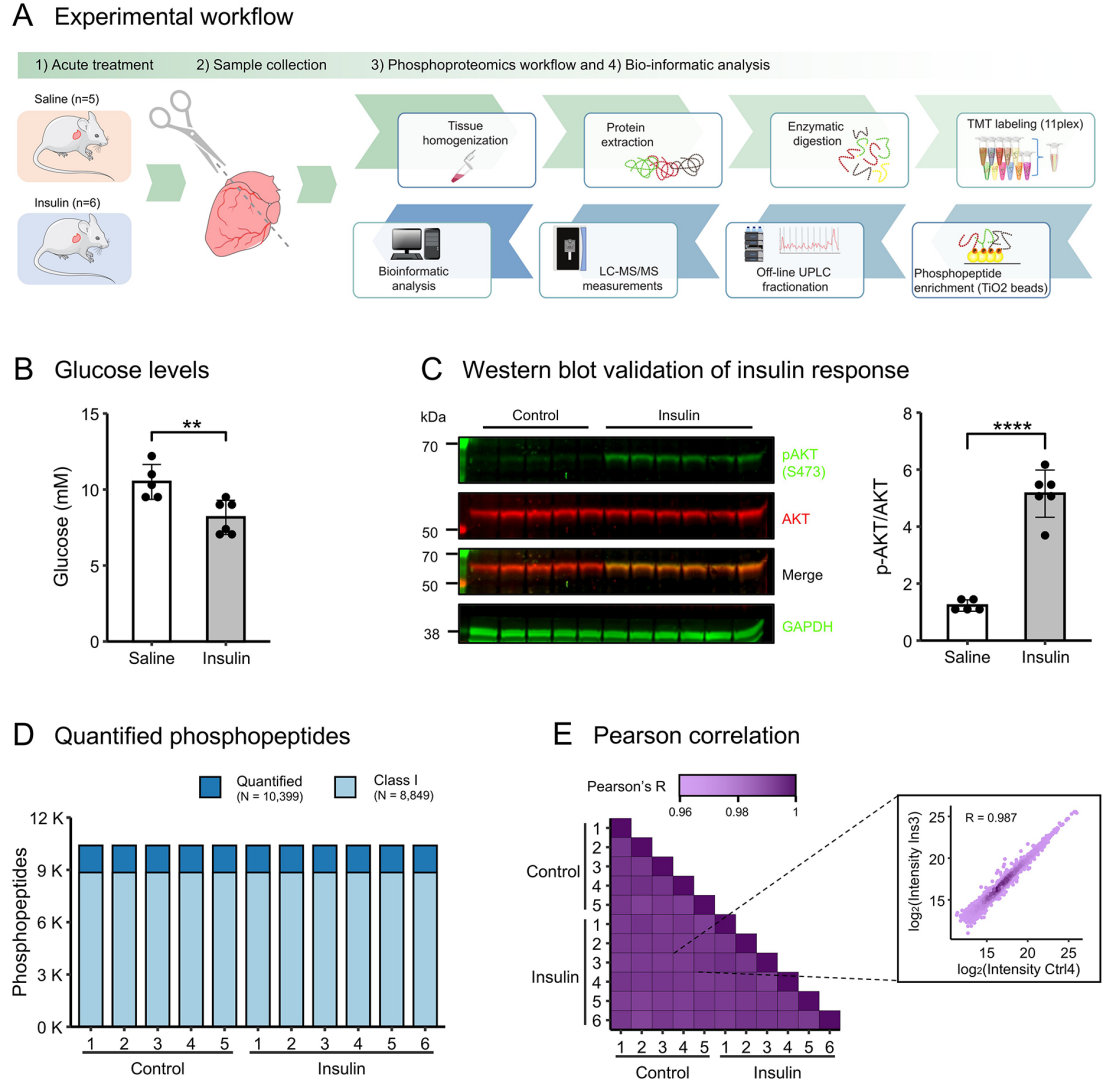
Induction of an insulin signaling response in cardiac tissue and phosphoproteomics data collection. **(A)** Schematic presentation of the experimental workflow: C57BL6/JRj male mice were i.v. injected with saline (*n* = 5) or insulin (*n* = 6, 0.75U/Kg BW) via the vena cava. Cardiac ventricles were collected 10 min post-injection. Proteins extracted from the cardiac tissue were enzymatically digested and labeled with TMT reagents prior to enrichment of phosphorylated peptides. Samples were fractionated on an offline UPLC system prior to measurement by tandem mass spectrometry. **(B)** Glucose levels were measured in the mice by tail-bleeding before and after treatment. No significant differences in plasma glucose levels were observed before injection (not shown, *p* = 0.78). Data represents mean ± standard deviation (SD) 10 min after saline/insulin bolus. Two-tailed Mann-Whitney test **p ≤ 0.01 (*p* = 0.0087). **(C)** Western blot analysis of phosphorylated AKT (p-AKT S473) and total AKT in response to saline (*n* = 5) or insulin (*n* = 6) stimulation in ventricle tissue lysates. Quantification of p-AKT/total AKT ratio was determined by Image Studio Lite software. A representative Western blot is shown at the left. Data represents mean ± SD. Independent t-test ****p ≤ 0.0001. **(D)** The number of phosphorylated peptides measured across samples. 10,399 phosphorylated peptides could be quantified across all samples. Of these, more than > 85% (8,849) had a phosphorylation event localized to a specific residue with site localization probability > 0.75 (class I). **(E)** Pairwise Pearson correlation coefficients for all measured phosphopeptide intensities. Insert shows an exemplary correlation plot. The average correlation coefficient is 0.989

We set out to study insulin-induced phosphorylation signal transduction in murine hearts in vivo. To this end, insulin (0.75U/Kg BW) or saline was administered through the inferior vena cava to anesthetized C57BL6/JRj male mice, followed by a 10-minute interval for insulin to exert its function (Fig. 1A). An insulin response was confirmed from plasma glucose measurements showing reduced glucose levels in insulin stimulated mice prior to tissue collection (*p* = 0.0087) (Fig. 1B). To further evaluate the insulin response in the cardiac tissue, immunoblot analysis was conducted on ventricular tissue lysates from the mice. This showed an augmentation in the phosphorylation at the S473 residue of the serine/threonine kinase Akt (Fig. 1C), which is an established indicator of Akt activation [24]. Upon verifying the insulin-induced signaling response in the cardiac tissue of mice stimulated by insulin, we proceeded with mass spectrometry-based quantitative phosphoproteomics measurements to conduct an examination of the cardiac insulin signaling response (Fig. 1A). We identified a total of 14,975 peptides, of which 72% were phosphorylated (Supplemental Fig. S1A-C). Among the phosphorylated peptides, 10,399 could be quantified across all samples (Fig. 1D), localizing to 3,232 unique proteins. Notably, phosphorylation events could be assigned to specific residues in 8,849 (85%) of the quantified peptides, signifying class I phosphorylation sites (Fig. 1D). The Pearson correlation coefficients of the measured phosphopeptide intensities, depicted in Fig. 1E, demonstrate an average Pearson correlation coefficient exceeding 0.98. This highlights the high technical reproducibility across all samples and attests to the precision of our mass spectrometry data. Principal component analysis (PCA) separated insulin-treated samples from control samples along the first three principal components, collectively explaining 42% of the variance observed in the dataset (Supplementary Fig. S1D). Hence, we have compiled an extensive phosphoproteomics dataset derived from cardiac tissue that captures the biological response elicited by insulin stimulation. The data for all quantified phosphorylation events are provided in Supplemental Table S1.

### Insulin-induced protein phosphorylation signal transduction in cardiac tissue

**Fig. 2:**
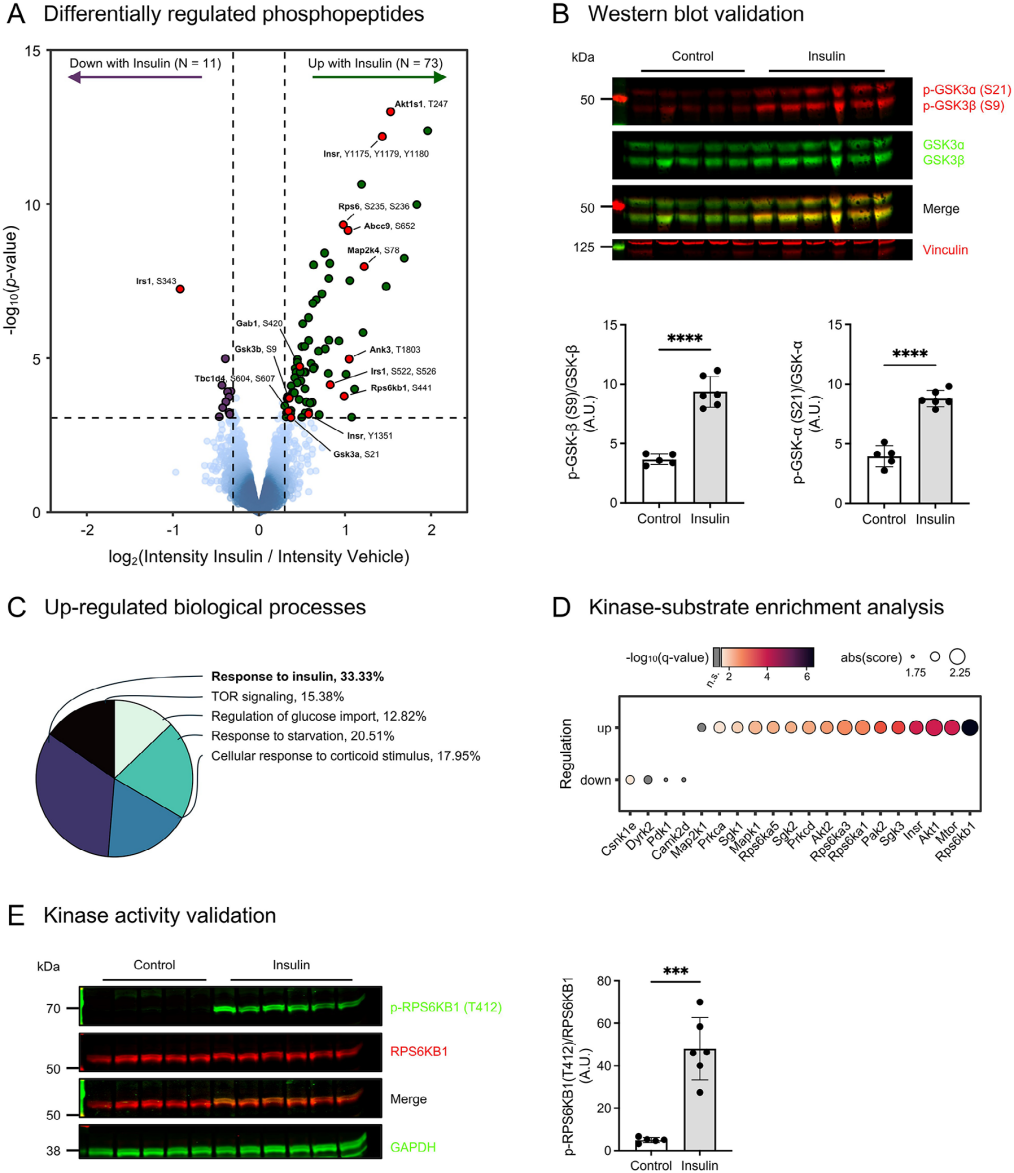
Global insulin-signaling response elicited in cardiac tissue. **(A)** Volcano plot showing differentially phosphorylated peptides after acute stimulation with insulin. The log_2_(fold change) is plotted against the –log_10_(p-value) for each phosphopeptide. Dark green dots indicate increased phosphorylation upon insulin stimulus compared to controls (logFC > 0.3 & adjusted p-value < 0.1), whereas dark purple dots represent decreased phosphorylation levels (logFC < -0.3 & adjusted p-value < 0.1). Selected phosphorylation sites are highlighted in red, such as Glycogen synthase kinase 3-alpha and beta (GSK3α^S21^, GSK3β^S9^) and Insulin receptor (Insr^Y1175, Y1179, Y1180^). **(B)** Validation of a target found as significantly regulated in the phosphoproteome analysis. Representative blot showing phospho-GSK3α^S21^ and GSK3β^S9^ levels in response to saline or insulin stimulation. The tissue lysates were used for phosphoproteome analysis (upper panel). Quantification of p-GSK/total GSK ratio for each isoform (lower panel). Data represents mean ± SD. Independent t-test ****p ≤ 0.0001. **(C)** Functional enrichment analysis of proteins harboring upregulated phosphorylation sites. The pie chart shows the percentage of representative upregulated biological processes in insulin treated samples. **(D)** Kinase-substrate enrichment analysis. The x-axis indicates the kinase gene and the y-axis the direction of regulation. Dots are scaled by the absolute enrichment score and colored by significance. **(E)** Western blot of top enriched kinase, Ribosomal Protein S6 Kinase B1 (Rps6kb1 also known as P70s6k). Phosphorylated Rps6kb1 on residue T412 in response to saline (*n* = 5) or insulin (*n* = 6) in ventricular tissue lysates (left panel). Quantification of p-Rps6kb1/total Rps6kb1 ratio (right panel). Data represents mean ± SD. Independent t-test ****p ≤ 0.0001. Source data is provided in Supplementary Table S1

Utilizing the quantitative phosphoproteomics dataset, we can assess the regulation of phosphorylation sites in response to insulin stimulation. To this end, we conducted differential abundance analysis of the measured phosphorylated peptides (Fig. 2A). Our analysis unveiled substantial signaling regulation in response to insulin stimulation, with 84 differentially regulated phosphorylated peptides. Specifically, 73 phosphorylation events were markedly upregulated (denoted in green), while 11 were significantly downregulated (denoted in purple) in the cardiac tissue following insulin stimulation. Comprehensive details regarding all significantly regulated phosphorylation sites are available in Supplementary Table S1. Included among the upregulated phosphorylation sites were known sites, such as tyrosine phosphorylation sites on the Insulin receptor (Insr^Y1175, Y1179, Y1180, Y1351^), in addition to phosphorylation sites on the adaptor protein Insulin receptor substrate protein 1 (Irs1^S522, S526^). Notably, Irs1 also harbored a site with down-regulated abundance (Irs1^S343^). Furthermore, several regulated phosphorylation sites were identified on kinases and proteins known to modulate kinase activity. These include glycogen synthase kinase-3 alpha (Gsk3α^S21^), glycogen synthase kinase-3 beta (Gsk3β^S9^), ribosomal protein S6 kinase beta-1 (Rps6kb1^S441^), 40S ribosomal protein S6-(Rps6^S235, S236, S240, S244^), and 5’-AMP-activated protein kinase catalytic subunit alpha-2 (Prkaa2^S377^), as well as the negative regulator of the mechanistic target of rapamycin complex 1 (mTORC1), Akt1s1^T247^. To corroborate the phosphoproteomics findings, we conducted immunoblot analysis for two of the regulated phosphorylation sites. This confirmed a significant elevation in the phosphorylation of Gsk3α^S21^ and Gsk3β^S9^ in hearts post insulin stimulation in vivo (Fig. 2B). Functional enrichment analysis of proteins exhibiting elevated phosphorylation levels following insulin stimulation highlighted the canonical response to insulin, TOR signaling, and responses to starvation and glucose import regulation (Fig. 2C, Supplementary Fig. S2). To predict the kinases involved in the cardiac insulin signaling response from the phosphoproteomics data, we performed enrichment analysis leveraging previously annotated kinase-substrate interactions. Our analysis implicated several kinases in the cardiac insulin response, including Rps6kb1, Akt1, Mtor, Rps6ka1, Sgk3, and Insr itself (Fig. 2D). A number of these kinases were found to have increased phosphorylation levels themselves, including Rps6kb1 (Fig. 2A). We validated the top finding from the analysis through immunoblotting, affirming that insulin stimulation induced a significant augmentation in the phosphorylation of T412 of the p70 S6 kinase, Rps6kb1 (Fig. 2E), which is known to activate the kinase [25]. Consequently, the primary insulin signaling response elicited in cardiac tissue by in vivo insulin stimulation underscores the canonical insulin signaling pathway. Detailed information about all regulated specific residues is provided in the Supplementary Table S1.

### Insulin stimulation induces canonical and cardiomyocyte-specific phosphorylation signaling responses

**Fig. 3:**
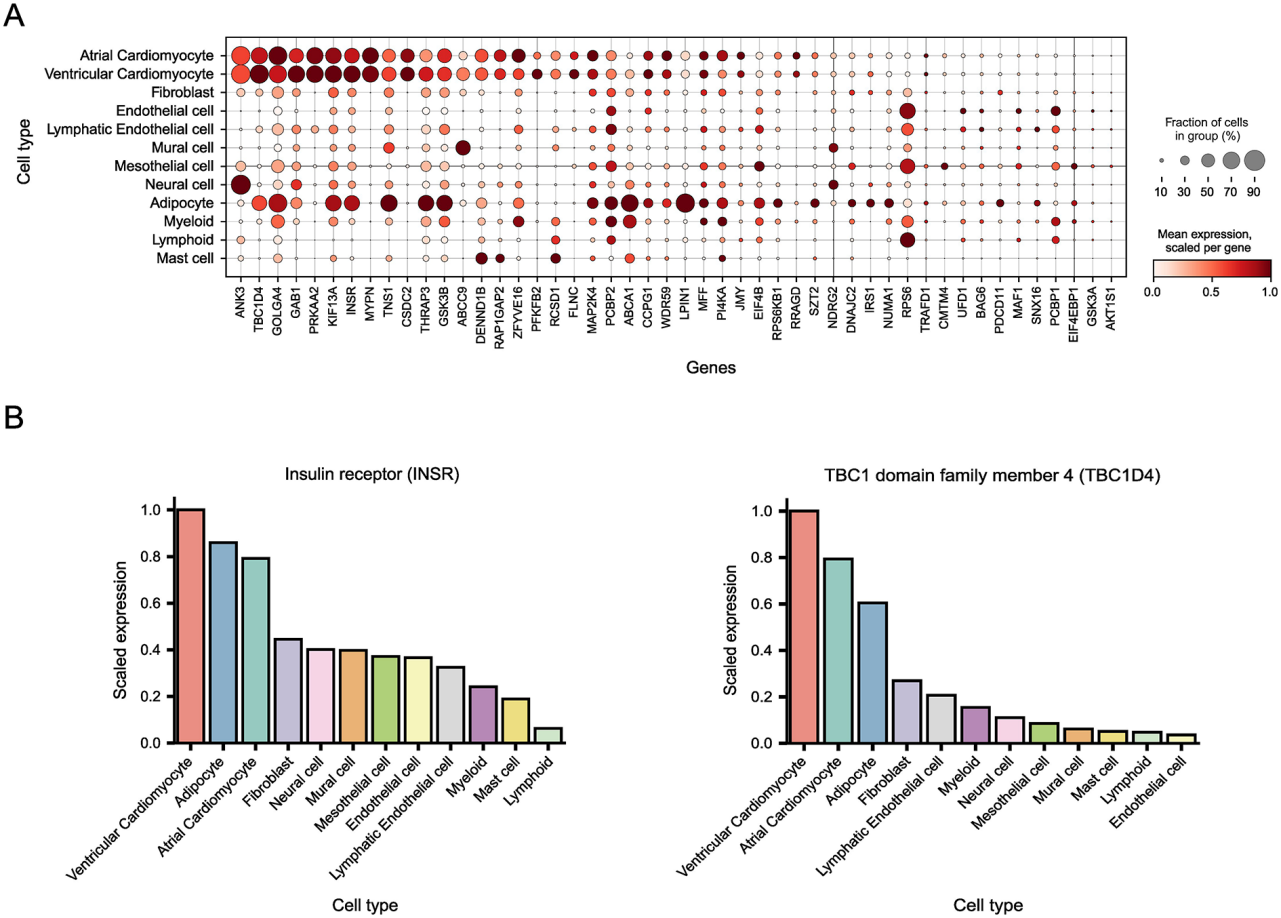
Cardiac insulin signaling response is mainly attributed to cardiomyocytes and adipocytes. **(A)** Cell-type inference from single cell RNA sequencing data. Dot plot representation of average expression levels and prevalence among cardiac cell types inferred from single-cell RNA sequencing data [26]. Shown are genes with significantly regulated phosphorylation events following insulin stimulation in the murine heart. The size of every dot indicates the fractions of cells (%) expressing the gene, the color depicts the scaled expression of the gene in each cell type. **(B)** Scaled expression of the insulin receptor (Insr, left) and TBC1 domain family member 4 (Tbc1d4) highlighting cardiomyocytes as the predominant cell types

**Fig. 4:**
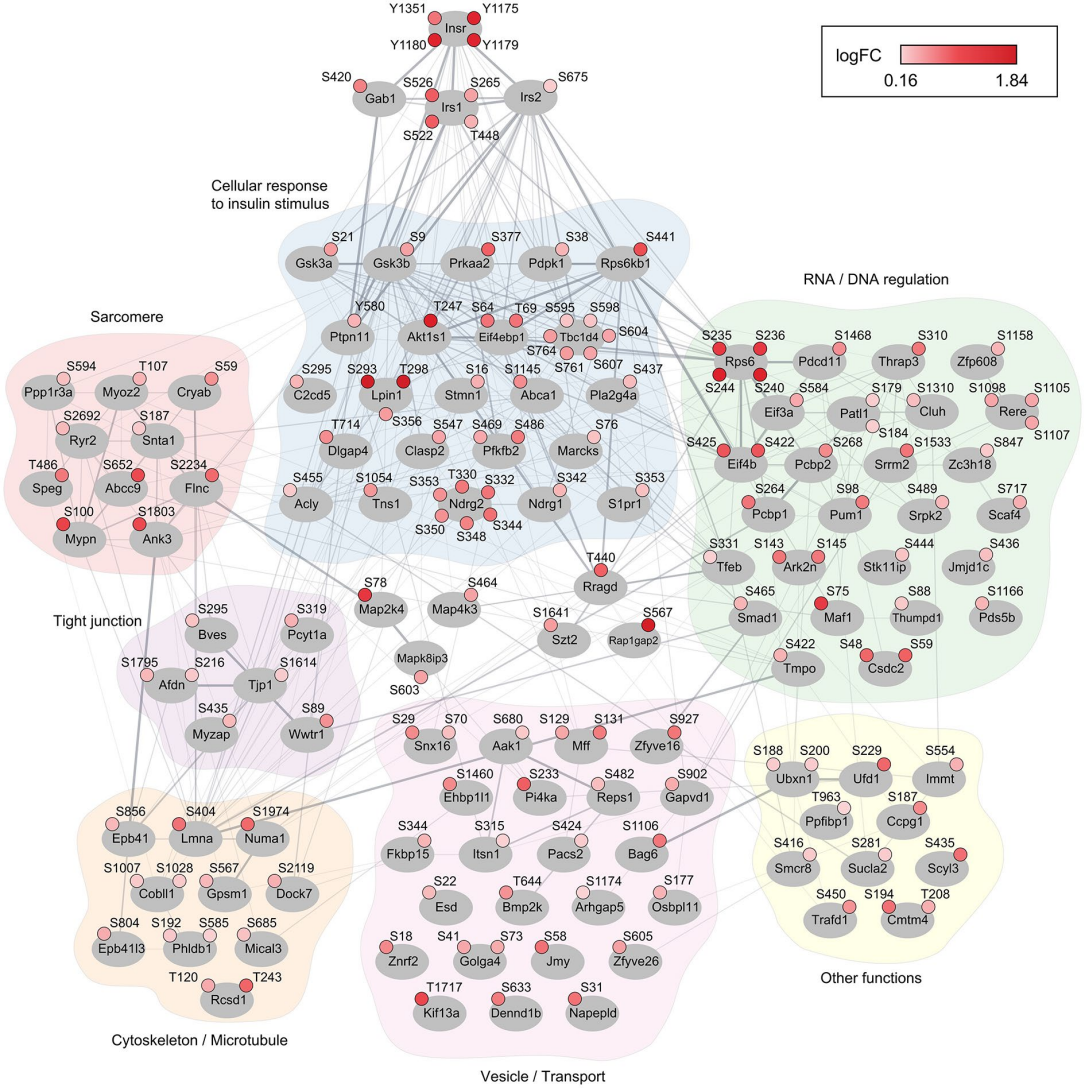
Graphical summary of the insulin signaling response in cardiac tissue. Graphical representation of the proteins encompassing the 150 most regulated phosphorylation sites measured in the cardiac tissue after 10 min of insulin stimulation. Proteins are represented by their gene name, and up-regulated phosphorylation sites are indicated by the regulated residue, the amino acid number, and log_2_(fold change). All phosphorylation sites are reported in Supplementary Table S1

Our analyses thus far have indicated a canonical insulin signaling response in cardiac tissue. However, the specific cardiac cells responsible for this response remain unidentified. To address this, we employed an informatics-based evaluation to discern the cardiac cell populations associated with proteins exhibiting upregulated phosphorylation sites. By intersecting proteins encompassing regulated phosphorylation sites with cardiac single-cell RNA sequencing data from the heart cell atlas v2 [26], we inferred that the predominant cell populations responding to insulin stimulation in the heart are cardiomyocytes and adipocytes (Fig. 3A). Importantly, in the heart, the cell population with the greatest expression level of the insulin receptor itself is the cardiomyocyte (Fig. 3B). Other highly regulated proteins presenting a similar expression pattern, with a predominant expression in cardiomyocytes, included Tbc1d4. Considering the specialized nature of cardiomyocytes and their unique protein expression profiles, we surmised that these cells also exhibit regulation beyond the canonical signaling pathway. The regulation of Abcc9, as shown in Fig. 2A, which is a regulatory subunit of the cardiac ATP-sensitive potassium (K_ATP_) channel, is supportive of this. To deconvolute the intricate phosphorylation response triggered by insulin stimulation in the heart, and to extend our understanding beyond the canonical signaling pathway, we assessed the biological function of the proteins that comprise the most regulated phosphorylation sites (Fig. 4). We generated a protein-protein interaction network and classified these proteins into functional groups. These were: (1) the canonical insulin signaling response, (2) RNA/DNA regulation, (3) sarcomeric proteins, (4) tight junction proteins, (5) cytoskeletal and microtubule proteins, and (6) vesicle and transport proteins. Figure 4 provides a graphical summary of the insulin signaling response triggered in cardiac tissue. Many of the identified phosphorylation sites are known to be regulated in other cellular systems following insulin stimulation. However, we also observed regulation on proteins with specialized functions in cardiomyocytes, such as Abcc9, Ryr2 and Ank3. Most of the regulated proteins in the canonical signaling response are involved in metabolic regulation. Tbc1d4, a protein heavily regulated, is one such protein. As depicted in the Fig. 4, multiple phosphorylation sites on Tbc1d4 exhibited increased abundances following insulin stimulation, including S595, S598, S604, S607, S761 and S764.

### Tbc1d4 is involved in Glut4 translocation and glucose uptake processes in cardiomyocytes

**Fig. 5:**
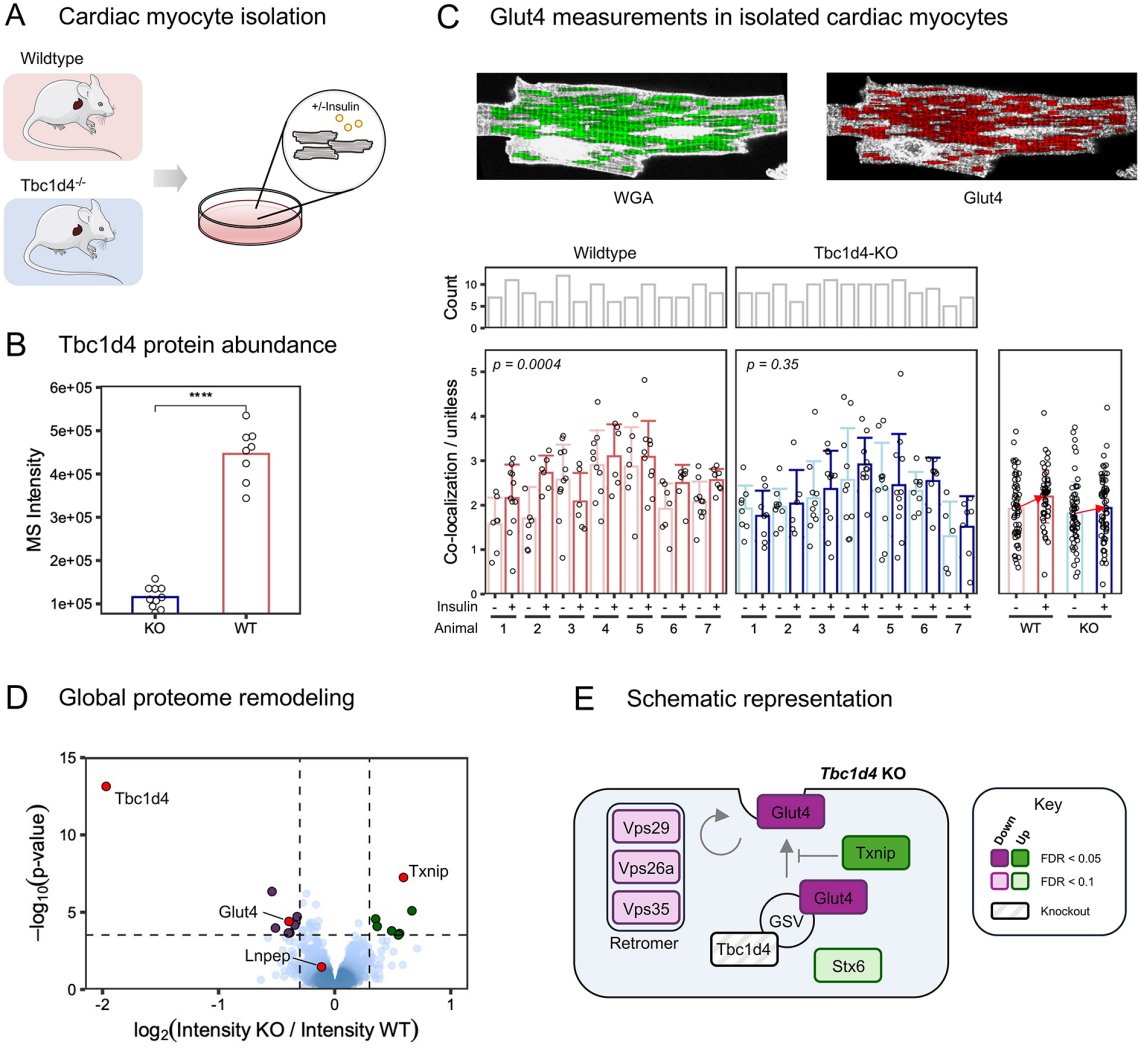
Tbc1d4 deficiency in cardiomyocytes affects insulin-induced Glut4 translocation and causes protein remodeling. **(A)** Schematic illustrating that cardiomyocytes were isolated from *Tbc1d4* knockout (KO) mice as well as from wildtype (WT) littermates. **(B)** Protein intensity of Tbc1d4 in cardiomyocytes isolated from WT and Tbc1d4 KO mice, evaluated by mass spectrometry-based proteomics. Tbc1d4 abundance is reduced 4-fold (**** adjusted p-value < 0.0001, BH correction). **(C)** Glut4 translocation to t-tubules upon insulin stimulation is shunted in Tbc1d4 deficient cardiomyocytes. Top: Fluorescence intensity from a representative cardiomyocyte co-stained for WGA (left) and Glut4 (right). Green regions are proximal to the t-tubules, and these regions were selected to assess Glut4 translocation (red). Bottom: Cardiomyocytes were isolated from *n* = 7 WT and *n* = 7 KO mice and treated with either insulin or vehicle followed by assessment for co-localization of Glut4 with the t-tubules. Single-cell co-localization data are clustered by genotype (WT vs. KO), grouped per mice, and subdivided by treatment (+/- insulin). Cell-specific data points are indicated by open circles, bar graphs show group means. Error bars represent the standard deviation of the mean. The plot to the right summarizes the genotype-specific data. The positive slopes of the arrows indicate enhanced co-localization of Glut4 with the t-tubules after insulin stimulation. This enhanced co-localization with insulin stimulus was attenuated in KO cardiomyocytes (*p* = 0.35) compared to WT (*p* = 0.0004 by compounded unidirectional Mann-Whitney U-tests). **(D)** Volcano plot visualization of differentially expressed proteins between KO and WT cardiomyocytes. The log_2_(fold change) is plotted against the -log_10_(p-value) for each protein. Dark green dots indicate significantly increased abundance in KO mice compared to WT (logFC > 0.3 & adjusted p-value < 0.05, BH-corrected), whereas dark purple dots represent significantly decreased abundance levels (logFC < -0.3 & adjusted p-value < 0.05, BH-corrected). **(E)** Visual representation of proteins involved in Glut4 translocation. Color-coded by direction of regulation and significance level

We identified Tbc1d4 as a major regulated protein in the heart following insulin stimulation (Fig. 4). Tbc1d4 plays a pivotal role in glucose uptake and Glut4 translocation in skeletal muscle, where Akt phosphorylation leads to inhibition of its RabGTPase activating function affecting Glut4 translocation to the plasma membrane and thereby glucose uptake [27]. The integration of our phosphoproteomics data with single-cell RNA expression data pointed to a predominant expression of Tbc1d4 in cardiomyocytes within the heart (Fig. 3B). Consequently, we sought to investigate how Tbc1d4 contributes to insulin signal transduction in cardiomyocytes. To this end, we employed a cardiac and skeletal muscle-specific *Tbc1d4* knockout (KO) model generated on a mixed background with a major component of C57BL/6NRj [28]. Cardiomyocytes were isolated from the hearts of the *Tbc1d4* KO mice and from their corresponding wildtype (WT) littermates (Supplementary Fig. S3). WT littermates were chosen as controls to ensure the same genetic background in both groups. We confirmed that protein abundance of Tbc1d4 was significantly diminished in cardiomyocytes from *Tbc1d4* KO mice compared to WT (Fig. 5B). To explore whether Tbc1d4 deficiency influences Glut4 translocation in response to insulin within cardiomyocytes, we assessed Glut4 localization in isolated adult cardiomyocytes from both WT and *Tbc1d4* KO mice utilizing high-resolution microscopy. Following isolation, cardiomyocytes underwent brief culturing and were stimulated with either saline or insulin, after which they were fixed and stained for Glut4. To delineate the plasma membrane and t-tubules of the rod-shaped cardiomyocytes, we co-stained the cells with wheat germ agglutinin (WGA) (Supplementary Fig. S4). As Glut4 preferentially translocates to t-tubules in cardiomyocytes in response to insulin stimulus [29], we quantified the intensity profile of Glut4 in relation to the t-tubules from the acquired images (Fig. 5C, Supplementary Fig. S4). This analysis identified increased co-localization of Glut4 with the t-tubules following insulin stimulation in cardiomyocytes from WT mice, indicating that Glut4 translocates to the t-tubules upon insulin stimulation in these cells. However, the insulin-induced effect on Glut4 translocation was attenuated in Tbc1d4-deficient cardiomyocytes (Fig. 5C). Thus, Tbc1d4 appears to be involved in the translocation of Glut4 to the sarcolemma in cardiomyocytes in response to insulin stimulation.

In genetic knockout models, compensatory protein remodeling is a common occurrence. To investigate the presence of such compensatory regulatory networks in Tbc1d4-deficient cardiomyocytes, we investigated proteome profiles from isolated cardiomyocytes using mass spectrometry-based quantitative proteomics (Supplementary Fig. S5). Our data encompassed measurements of 6,060 unique proteins from cardiomyocytes (WT and *Tbc1d4* KO), of which 5,156 were quantified by at least two distinct peptide spectrum matches (PSMs). Detailed information on all quantified proteins is provided in Supplementary Table S2. Pearson’s correlation coefficients surpassed 0.98 across all replicates, underscoring the robustness of our approach (Supplementary Fig. S5A-C). Evaluation by principal component analysis suggested that differences between the two genotypes were best represented along the fifth component, explaining 8% of the variation in the dataset (Supplementary Fig. S5B). We identified 15 proteins exhibiting significant differences between *Tbc1d4* KO and WT cardiomyocytes (BH-corrected p-value < 0.05, abs(logFC) > 0.3, Fig. 5D). Notably, Glut4 and thioredoxin-interacting protein (Txnip) were differentially expressed, where Glut4 had reduced protein abundance in the KO cardiomyocytes and Txnip exhibited an increase of more than 50% (Fig. 5E-F). We also observed dysregulation of proteins involved in Glut4 trafficking, including syntaxin-6 [30] and retromer proteins Vps26a, Vps29, and Vps35 (BH-corrected p-value < 0.1, Supplementary Fig. S5) [31]. Thus, the compensatory protein remodeling consequent to Tbc1d4 deficiency entails proteins involved in the functions which Tbc1d4 is known for in skeletal muscle. Collectively, our findings support the involvement of Tbc1d4 in the insulin signaling response in cardiomyocytes, with a specific emphasis on glucose uptake processes.

### The insulin signaling response elicited in isolated cardiomyocytes is preserved in Tbc1d4 deficient cardiomyocytes

**Fig. 6:**
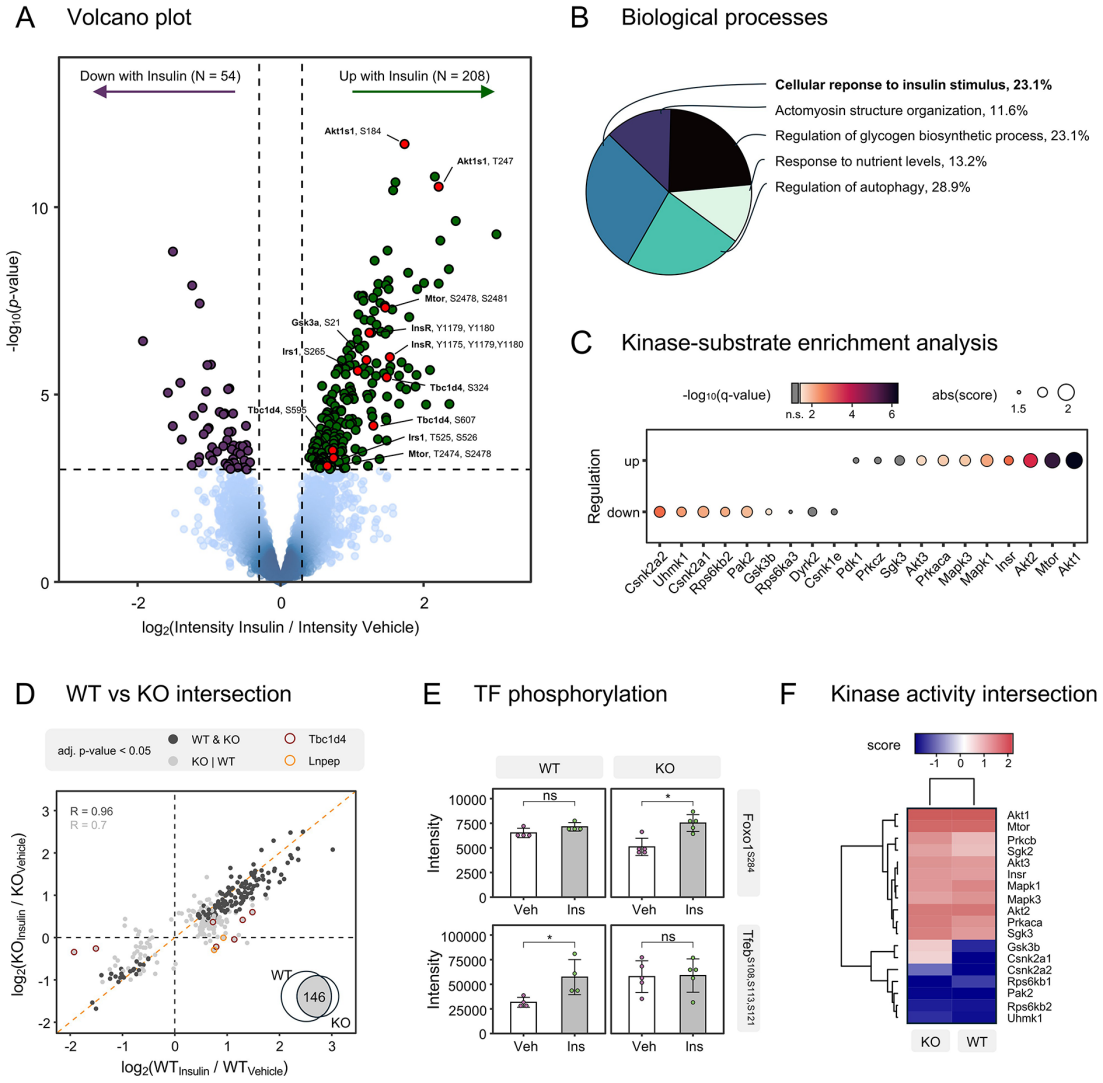
Phosphoproteomics profiling of the insulin signaling response in adult murine cardiomyocytes. (**A)** Volcano plot visualization of differentially phosphorylated peptides upon insulin stimulation in WT cardiomyocytes. The log_2_(fold change) is plotted against the–log_10_(p-value) for each phosphopeptide. Dark green dots indicate significantly increased phosphorylation upon insulin stimulus compared to controls (logFC > 0.3 & adjusted p-value < 0.05, BH-corrected), whereas dark purple dots represent significantly decreased phosphorylation levels (logFC < -0.3 & adjusted p-value < 0.05, BH-corrected). Representative phosphorylation sites, such as Insr^Y1175, Y1179, Y1180^, are highlighted in red. **(B)** Functional enrichment analysis of proteins harbouring significantly upregulated phosphorylation sites. The pie chart shows the percentage of representative upregulated biological processes in insulin treated samples. **(C)** Kinase-substrate enrichment analysis. The x-axis indicates the kinase gene and the y-axis the direction of regulation. Dots are scaled by the absolute enrichment score and coloured by significance. (**D)** Fold-change correlation among insulin-regulated phosphorylated peptides with significance (adjusted p-value < 0.05, BH-corrected) in at least one condition (WT or KO). Phosphorylation events with significance in WT and KO cardiomyocytes are highlighted in dark grey, whereas those with significance in only one condition are coloured in light grey. Phosphopeptides localizing to Tbc1d4 or Lnpep are highlighted in dark red and orange circles, respectively. Venn diagram showing the intersection size of significantly regulated phosphopeptides. (**E)** Differential impact of insulin stimulation on transcription factor (TF) phosphorylation. Intensities of phophopeptides Foxo1^S284^ and Tfeb^S108, S113, S121^ are depicted as bar charts showing the mean abundance for vehicle (Veh) and insulin (Ins) treated cardiomyocytes, separated by genotype (WT vs. KO). Error bars represent mean standard deviation, adjusted p-values are derived from limma(* *p* < 0.05). (**F)** Heatmap representation of kinase enrichment scores upon insulin stimulation of WT and Tbc1d4 KO cardiomyocytes.

Our phosphoproteomic analysis of tissue samples indicated that the insulin signaling response is primarily associated with a signal in cardiomyocytes. Considering the crucial role of cardiomyocytes in cardiac function, it becomes particularly important to explore the insulin signaling response within these cells. To this end, we isolated adult murine cardiomyocytes from ventricles and examined the insulin signaling response using mass spectrometry-based phosphoproteomics. Upon subjecting the isolated cardiomyocytes to either insulin (10nM) or saline for 30 min, we proceeded to extract proteins and implement a tandem mass tag (TMT)-multiplexed high-resolution proteomics and phosphoproteomics workflow (Supplementary Fig. S3). This approach enabled the quantification of 12,739 phosphorylated peptides, with site-specific phosphorylation assignments available for 8,247 (Supplementary Fig. S6A-C). The stimulation of cardiomyocytes with insulin led to the regulation of 262 phosphorylation events (Fig. 6A). Specifically, we noted increased phosphorylation events on the Insulin receptor (Insr^Y1175, Y1179, Y1180^), insulin receptor substrate (Irs1^S265^), proteins downstream of the insulin receptor (Akt1^T308^, Akt1s1^T247, S184^, Mtor^S2478, S2481^ Eif4b^S424, S425^, Rps6kb1^S447, S452^), and proteins related to glucose metabolism (Gsk3α^S21^, Gsk3β^S9^, Tbc1d4^S324, S607^), myofilaments (Mybpc3^S273, S307^), actin regulation (Fhod3^S345^, Mypn^S1281^, Flnc^S2234^), ion channel regulation (Kcnj11^Y12^) and proteins involved in trafficking and localization (Ralgapa2^T715^) and transcriptional regulators (Foxo3^S252^ and Foxo1^S253^) (Fig. 6A, Supplementary Table S3). Immunoblot analysis confirmed a significant elevation in the phosphorylation of Gsk3α^S21^, Gsk3β^S9^ and Akt^S473^ in isolated cardiomyocytes upon insulin stimulation (Supplementary Fig. S3). Proteins regulated by insulin were strongly enriched for biological processes “response to insulin”, and “glycogen metabolic processes”, “autophagy” and “actomyosin structure” (Fig. 6B). Kinase substrate enrichment analysis of the data suggested that these regulations are mediated by activation of Akt, Mtor, Mapk1 and Insr kinases (Fig. 6C). Among the various phosphorylation events, Akt1^T308^ and Mtor^S2478/S2481^ emerged as critical signaling hubs, e.g. modulating Tbc1d4 as a regulator of Glut4 trafficking. Phosphoproteome analysis of cardiomyocytes highlighted how insulin regulates phosphorylation of proteins implicated in glucose metabolism (Fig. 6B). Additionally, both bulk heart- and cardiomyocyte specific phosphoproteomes showed that Tbc1d4 is heavily regulated following insulin stimulation (Figs. 3 and 6A). This raises the question whether Tbc1d4 is involved in orchestrating parts of the phosphorylation signaling network in cardiomyocytes. To test this, we evaluated quantitative phosphoproteomics measurements of cardiomyocytes from the *Tbc1d4* KO mice and investigated the signaling response elicited upon insulin stimulation (Supplementary Fig. S6E). We found that deficiency of Tbc1d4 did not alter the overall phosphorylation response to insulin (Fig. 6D), with a high correlation of phosphopeptide regulation between insulin-treated WT and *Tbc1d4* KO cardiomyocytes (Pearson’s *R* = 0.89, adjusted-*P*-value ≤ 0.05 in either condition). Specifically, 146 phosphorylated peptides exhibited concordant changes in response to insulin in both conditions, with an overall Pearson correlation of 0.96. Most notable differences were dominated by phosphorylation changes on the Tbc1d4 protein, with the KO mice showing no significant insulin-induced phosphorylation events. The protein leucyl/cystinyl aminopeptidase (Lnpep), also known as insulin-regulated aminopeptidase (IRAP) and a cargo protein of Glut4 storage vesicles [32, 33], exhibited reduced phosphorylation following insulin stimulus in Tbc1d4 deficient cardiomyocytes (Fig. 6D). We also observed differential phosphorylation levels on transcription factors, including Foxo1^S284^ and Tfeb^S108, S113, S121^ (Fig. 6E). In line with the high correlation of phosphorylation fold changes, kinase-substrate enrichment analysis revealed similar kinase activation in Tbc1d4 deficient cardiomyocytes and in wildtype cardiomyocytes following insulin stimulation (Fig. 6F). Collectively, our data supports that Tbc1d4 is a greatly regulated protein in the insulin signaling response in cardiomyocytes, yet it has little influence on the global phosphorylation signaling network elicited upon insulin stimulation in cardiomyocytes. Details for all phosphorylation events in the Tbc1d4 deficient cardiomyocytes are provided in Supplementary Table S3.

**Fig. 7:**
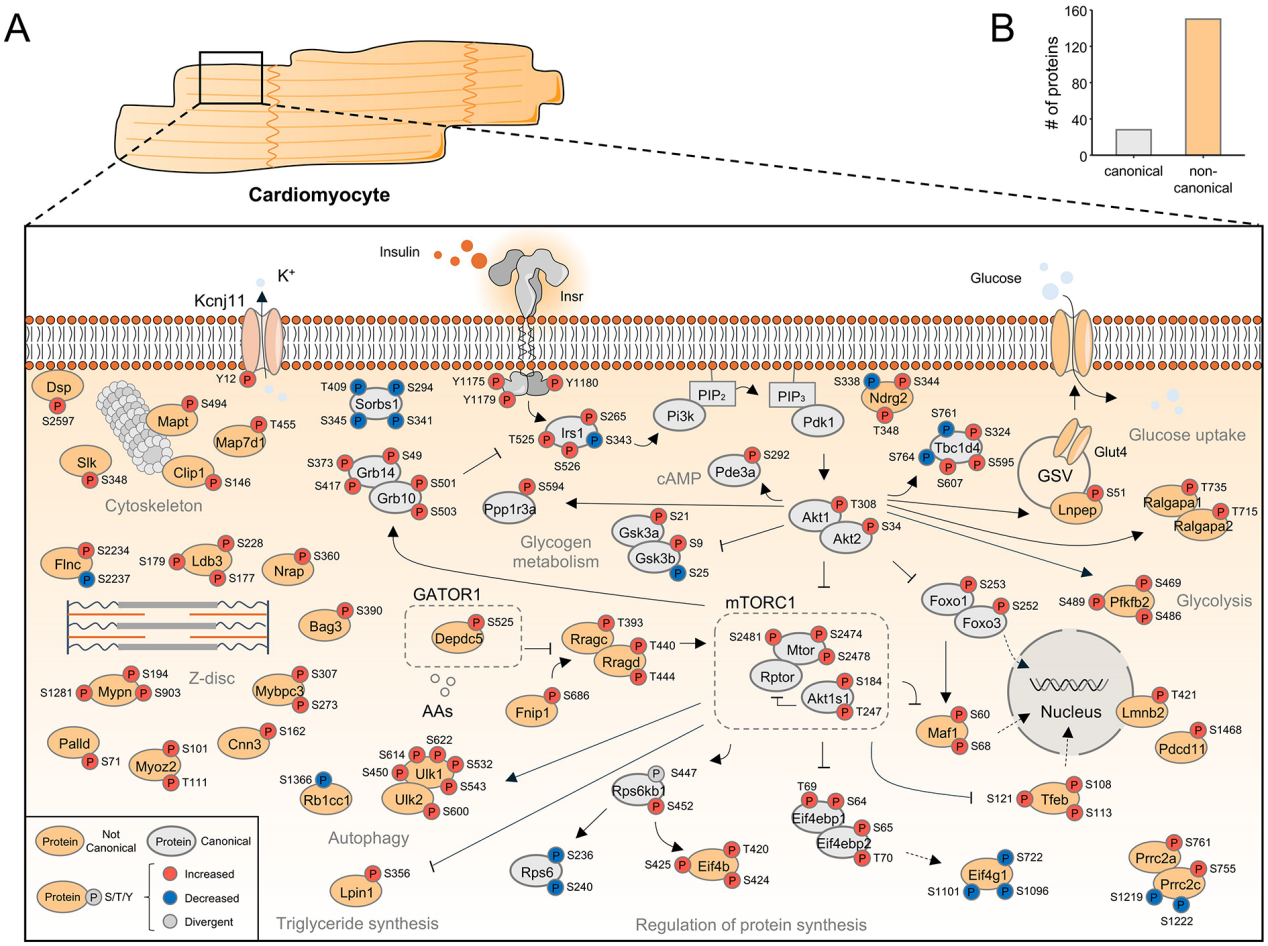
Graphical summary of the insulin signaling response in isolated adult murine cardiomyocytes. **(A)** Graphical representation of the phosphorylation response elicited by insulin stimulation in isolated cardiomyocytes. Proteins are represented by their gene name and classified into canonical and non-canonical insulin signaling. Regulated phosphorylation sites are indicated by the regulated residue, the amino acid number, and color-coded by direction of regulation. **(B)** Bar graph showing the proportion of canonical and non-canonical phospho-proteins. Details on all phosphorylation sites are provided in Supplementary Table S3

To better represent the global phosphorylation response in cardiomyocytes following insulin stimulation, we curated a graphical summary showcasing proteins with significant phosphorylation events in WT cardiomyocytes (P_adj_ < 0.05, log_2_ fold change > 0.3). Figure 7A delineates the specific residues regulated following insulin stimulation. Phosphorylation cascades initiating from the insulin receptor propagate downstream via recruitment of Irs1, activation of Akt kinase and the mTORC1 complex, to proteins with diverse biological functions such as transcription (Tfeb, Foxo1/3, Maf1), protein synthesis (Rps6, Eif4b, Eif4ebp1/2) and Glut4 translocation (Tbc1d4, Lnpep). Notably, many phosphorylation events localized to proteins not associated with the canonical insulin response (Fig. 7A, B). For instance, we demonstrate increased phosphorylation on myofilament proteins (Mybpc3, Mypn, Myoz2) and ion channels (Kcnj11) upon insulin stimulation. In fact, only 14% of the proteins harboring regulated phosphorylation events were found in a curated gene set of consensus insulin signaling. Taken together, these findings underscore the complexity of the insulin-regulated phosphorylation landscape and highlight how our unbiased global phosphoproteomic approach provides a comprehensive resource extending beyond canonical insulin signaling.

## Discussion

The present study deepens our understanding of the mechanisms through which insulin signaling influences cardiac function. We employed quantitative phosphoproteomics to decipher the precise phosphorylation events orchestrating the signaling response within cardiac tissue and cardiomyocytes. The substantial changes in phosphorylation underscore the importance of this process for modulating cardiac dynamics. Our cardiac tissue phosphoproteome data revealed regulation of proteins known to be part of the canonical insulin response, where we identified specific residues that are phosphorylated following insulin stimulation, adding detail to our understanding of insulin’s molecular regulation in the heart. Phosphorylation changes were found on the insulin receptor substrate proteins, Irs1 and Irs2, as well as on Shc1, which activate the phosphoinositide-3-kinase (PI3K), protein kinase B (Akt), and kinases of the Mapk family, respectively. Insulin is a potent Akt activator in the heart [34], a finding also supported by our data. Akt triggers the subsequent activation of glycogen synthase kinase 3 (Gsk3) and mechanistic target of rapamycin (Mtor), which also manifests from our phosphoproteome analysis. We observed the regulation of numerous other proteins, covering those involved in vesicle transport and regulations specific to cardiac tissue, suggesting unique roles for insulin signaling in cardiomyocytes. The latter includes regulation of ion channels [35], which is also supported by our data, where we found phosphorylation of Kcnj11^Y12^, Abcc9^S652^ and Ryr2^S2692^. In addition, we observed regulation of proteins such as Pde3a^S310^ and Prkaca^S339^, consistent with reports about cross-talk between insulin and β-adrenergic pathways [36, 37].

Our cardiac tissue-centric phosphoproteome dataset provided a global overview of specific phosphorylation sites on cardiac proteins regulated upon insulin stimulation. By administering insulin directly into the vena cava, we ensured that insulin reached the target organ first, allowing us to evaluate the cardiac signaling response while bypassing the variability associated with subcutaneous absorption and glucose challenge designs. Our approach aligns with previous investigations [38–40] and provides a focused assessment of insulin’s direct impact on the heart. However, the cellular identities of the response cannot be deciphered from this approach. Intersection of our tissue phosphoproteome data with single nuclei RNA sequencing data [26] suggested that the response was predominantly coupled to an expression profile matching cardiomyocytes and adipocytes. Given that cardiomyocytes are the primary cell type responsible for cardiac function and are much more abundant than adipocytes in the heart, we next evaluated the signaling response in primary cardiomyocytes. We isolated adult murine cardiomyocytes, exposed them to saline or insulin, and pursued measurements of the phosphoproteome. From this analysis, we identified phosphorylation changes on many proteins known to be part of the canonical signaling pathway (e.g., Irs1^S265^, Akt1^T308^, Akt1s1^S184, T247^, Mtor^S2478, S2481^, Gsk3a^S21^, Gsk3b^S9^, Rps6^S236, S240^, Rps6kb1^S452^), but also regulations specific for the cell type (e.g., Mybpc3^S307^ and Dsp^T59, S2597^).

Our study highlights Tbc1d4, a Rab-GTPase activating protein, as a focal point in cardiac insulin signaling. Both cardiac tissue and cardiomyocyte-specific phosphoproteomes showed extensive Tbc1d4 regulation post-insulin stimulation. Tbc1d4 modulates insulin-induced glucose uptake in adipocyte cells [41] and skeletal muscles [42], by controlling Glut4 translocation from cytosolic storage vesicles to the plasma membrane [12, 43–45]. Genetic variants in *TBC1D4* are associated with insulin resistance and type-2 diabetes in the Greenlandic Inuit population [46, 47], and regulation of TBC1D4 is impaired in skeletal muscle of people with type 2 diabetes [48]. Recent studies show that Tbc1d4 is also essential for insulin-stimulated glucose uptake and metabolic flexibility in the heart [49]. *Tbc1d4* KO reduced insulin-stimulated glucose uptake in cardiac tissue [49] and decreased Glut4 expression [49, 50]. Other studies have reported a direct link between Tbc1d4 phosphorylation and cardiac electrophysiology [51]. In cardiomyocytes, insulin induces Glut4 translocation towards sarcolemmal t-tubules [29], and Akt phosphorylates Tbc1d4 promoting Glut4 translocation [52].

Using cardiac and skeletal muscle-specific *Tbc1d4* KO mice [28], we examined the insulin signaling response in Tbc1d4-deficient cardiomyocytes. We studied Glut4 translocation towards the t-tubules following insulin stimulation and found that Tbc1d4 deficiency attenuated insulin-induced translocation of Glut4. This is consistent with earlier reports indicating that glucose uptake is abolished in the cardiac tissue of *Tbc1d4* KO mice [49]. Notably, analysis of the same tissue also revealed an increase in cardiac glycogen content [49], indicative of altered insulin-mediated glucose metabolism due to *Tbc1d4* deficiency. These findings emphasize Tbc1d4’s role in insulin-induced substrate utilization adaptations in cardiomyocytes. Despite Tbc1d4’s importance for these processes, we found that it has a limited impact on the insulin signaling network. Phosphoproteomes from both control and *Tbc1d4* KO cardiomyocytes responded similarly to insulin stimulation, suggesting the overall insulin-signaling pathway is preserved in Tbc1d4 deficient cardiomyocytes.

There were a few notable differences in the signaling response in cardiomyocytes deficient in Tbc1d4. Forkhead box protein O1 (Foxo1), a transcription factor associated with apoptosis, oxidative stress, and metabolic regulation [53], is reported to regulate Glut4 expression [54]. Increased Foxo1 transcriptional activity has been linked to cardiac dysfunction [55] and diabetic cardiomyopathy [56]. Foxo1 activation has been associated with Akt hyperactivation and attenuation of insulin signaling due to phosphatase inhibition [57]. In *Tbc1d4* KO cardiomyocytes, Foxo1^S284^ presented with a greater phosphorylation change upon insulin stimulation than in control cells, suggesting altered Foxo1 transcriptional regulation that could influence proteins involved in glucose homeostasis.

Our proteome analysis of *Tbc1d4* KO cardiomyocytes versus WT cardiomyocytes revealed a significant downregulation of Glut4, which has been previously reported in hearts of *Tbc1d4* KO mice [49, 50] and associated with decreased glucose uptake. In isolated cardiomyocytes, this reduced Glut4 expression coincided with a significant increase in Thioredoxin-interacting protein (Txnip). In the liver, Txnip expression is regulated by Foxo1 [58]. Txnip is involved in various cellular processes including anti-oxidative response, glucose metabolism, and autophagy, and Txnip regulation facilitates Glut4 endocytosis [59]. Intriguingly, Txnip is associated with hyperglycemia and diabetic cardiomyopathy [60–62]. The observed changes in Txnip and Glut4 protein abundances, altered Foxo1^S284^ signaling, and attenuated Glut4 translocation to cardiomyocyte t-tubules in Tbc1d4 deficient cardiomyocytes underscore the crucial role of Tbc1d4 in insulin-regulated glucose uptake in cardiomyocytes. Alteration in this axis could provide a pathological substrate that exacerbates cardiac function. Indeed, a recent study showed that Tbc1d4 deficiency worsened cardiac damage following myocardial infarction [49].

Under pathological conditions, such as diabetes or heart failure, the transduction and action of insulin signals are modified. These states, characterized by either insulin resistance or hyperinsulinemia, lead to adaptations in cardiac insulin signaling. Specifically, myocardial insulin resistance is associated with impaired insulin-mediated glucose uptake, reduced mitochondrial oxidative capacity, and a decreased Glut4 content. Adaptations to insulin signaling in obese or diabetic patients are believed to contribute to their increased risk of heart failure. Moreover, myocardial insulin signaling is also compromised in heart failure [63]. However, the mechanisms and physiological impacts of these impaired insulin signals in hearts under such pathological states are yet to be fully understood. Elucidating the signal transduction mechanisms involved in the cardiac insulin signaling response could potentially pave the way for identifying therapeutic approaches to treat cardiac diseases associated with insulin resistance. Future research should aim to detail how the insulin signaling network becomes dysregulated under pathological conditions. A crucial first step in this direction is to establish the baseline map of the insulin signaling response in cardiac tissue. This study represents such a first step by outlining the specific phosphorylation sites regulated in the heart and in cardiomyocytes upon insulin stimulation.

### Supplementary Information

Supplementary Table S1. List of phosphopeptides quantified upon acute insulin stimulation in murine cardiac tissue. Results of functional enrichment analyses.

Supplementary Table S2. List of proteins identified and quantified in isolated cardiomyocytes.

Supplementary Table S3. List of phosphopeptides quantified upon insulin stimulation in isolated cardiomyocytes. Results of functional enrichment analyses.

Supplementary Figure S1. Quality control for bulk phosphoproteomics data.

Supplementary Figure S2. Functional enrichment analysis.

Supplementary Figure S3. Proteomic and phosphoproteomic analysis of Tbc1d4-deficiency.

Supplementary Figure S4. Fluorescence imaging-based single-cell co-localization data processing.

Supplementary Figure S5. Quality control of Tbc1d4 KO and WT cardiomyocyte proteome measurements.

Supplementary Figure S6. Quality control of Tbc1d4 KO and WT cardiomyocyte phosphoproteome measurements.

Supplementary Material & Methods.

## Data Availability

Data is provided within the manuscript or supplementary information files. The mass spectrometry proteomics data have been deposited to the ProteomeXchange Consortium via the PRIDE partner repository with the dataset identifier PXD050545.
