# Supplementary figures and images for: In-depth phosphoproteomic profiling of the insulin signaling response in heart tissue and cardiomyocytes unveils canonical and specialized regulation
Source: Cardiovasc Diabetol. 2024 Jul 18;23:258. doi: 10.1186/s12933-024-02338-4 (PMC11264841; doi:10.1186/s12933-024-02338-4)

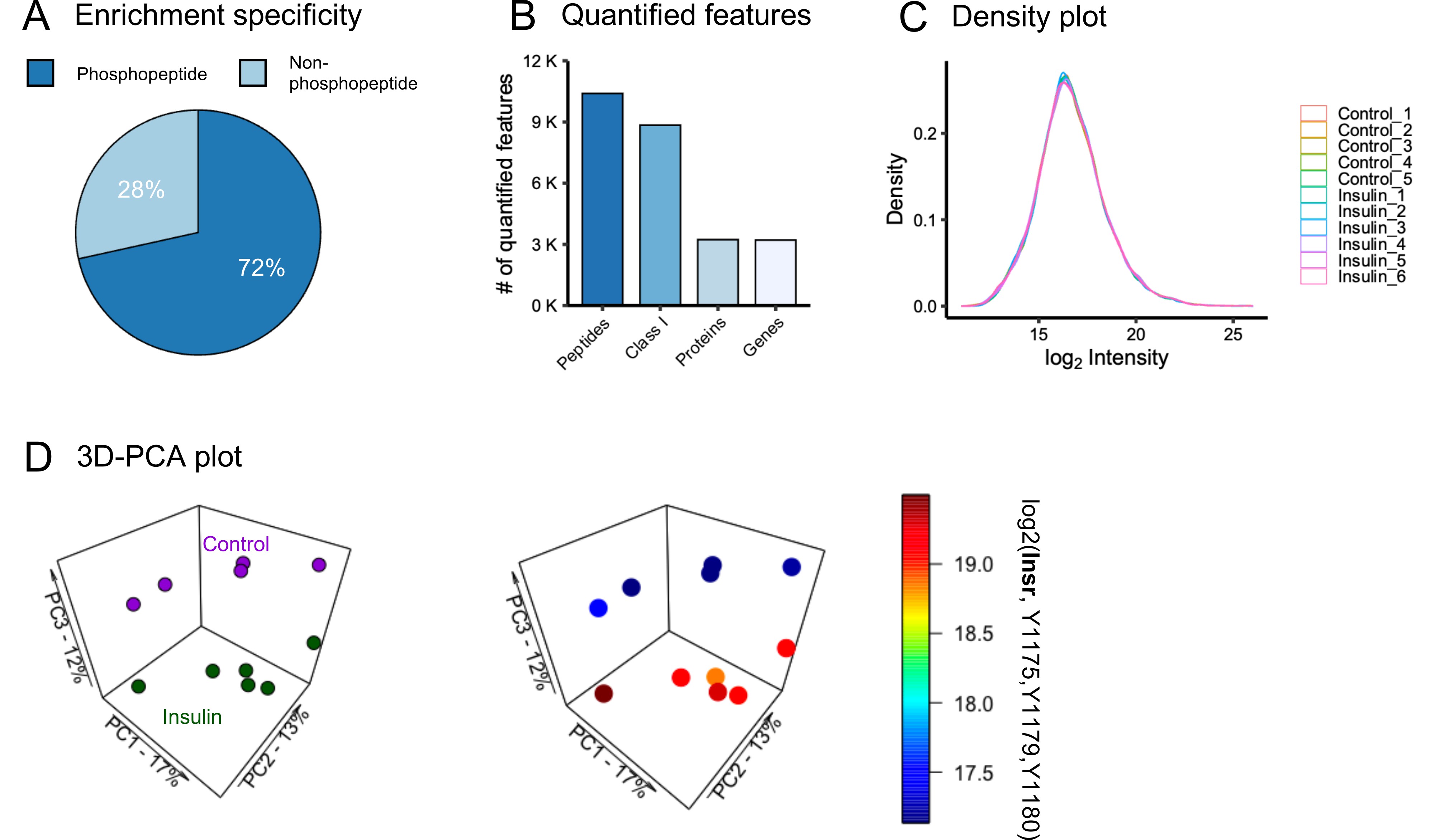

Supplement: Supplementary file 4 — Supplementary Figure S1. Quality control for bulk phosphoproteomics data. [file 12933_2024_2338_MOESM4_ESM.jpg]

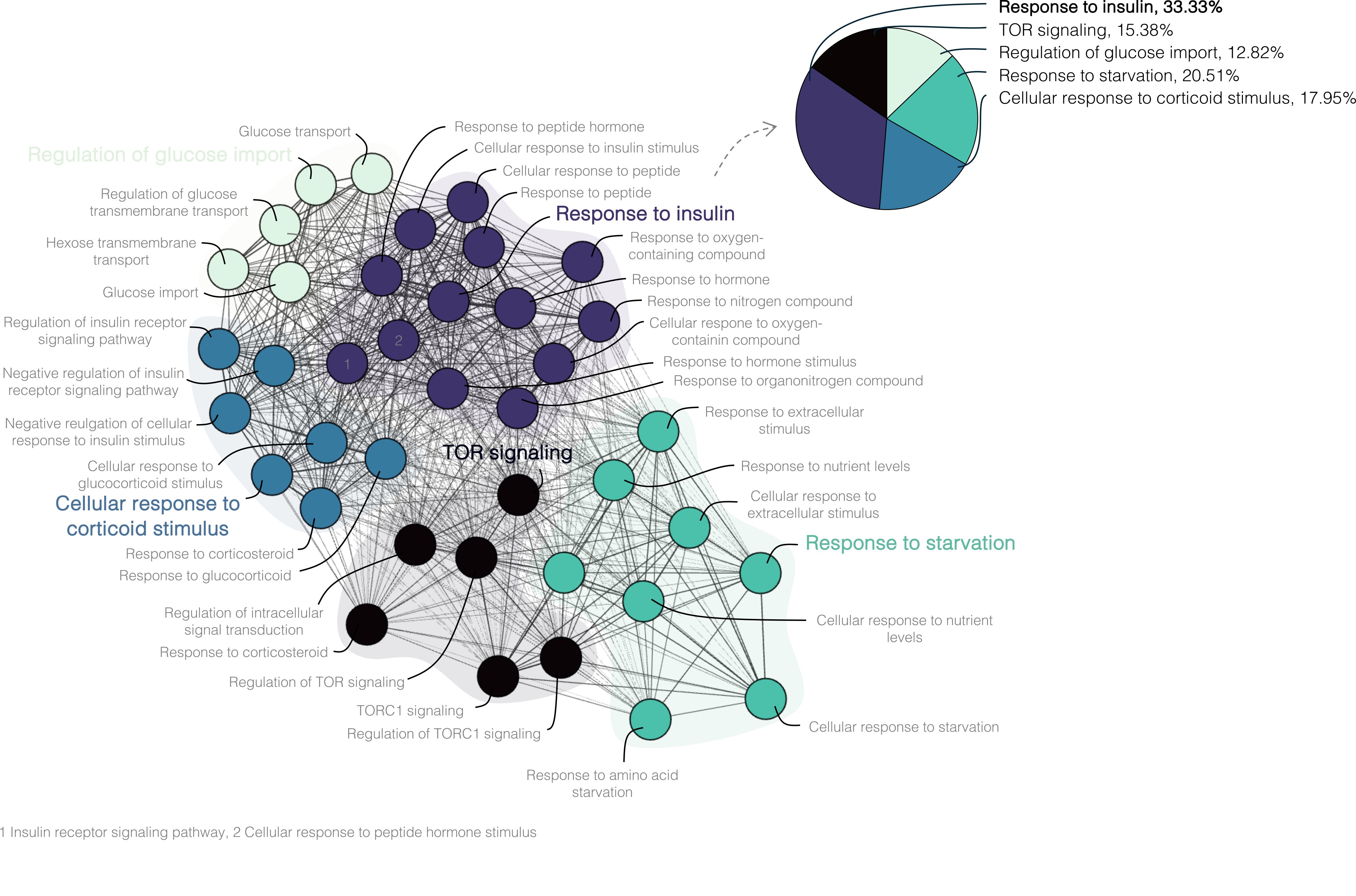

Supplement: Supplementary file 5 — Supplementary Figure S2. Functional enrichment analysis. [file 12933_2024_2338_MOESM5_ESM.jpg]

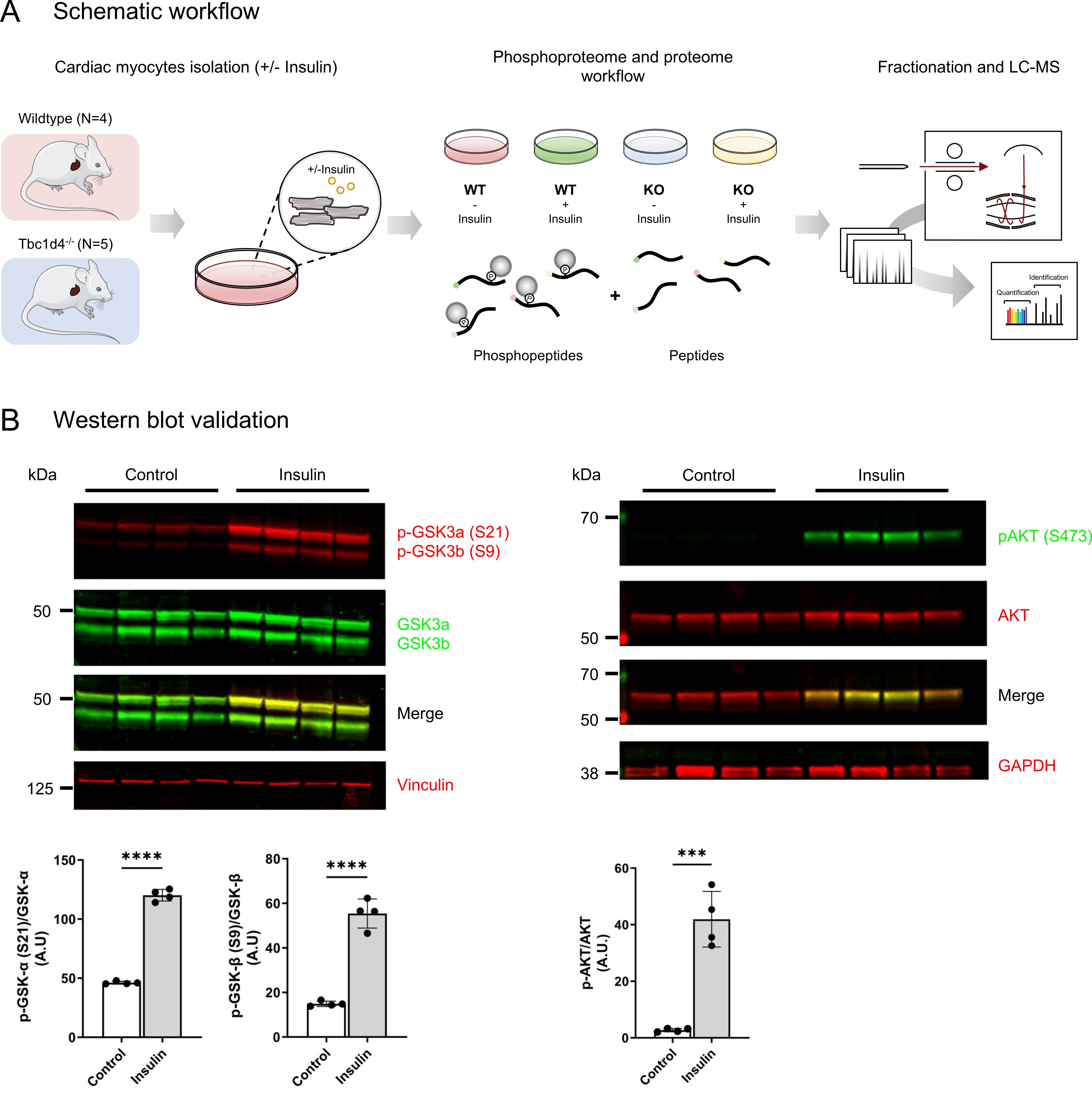

Supplement: Supplementary file 6 — Supplementary Figure S3. Proteomic and phosphoproteomic analysis of Tbc1d4-deficiency. [file 12933_2024_2338_MOESM6_ESM.jpg]

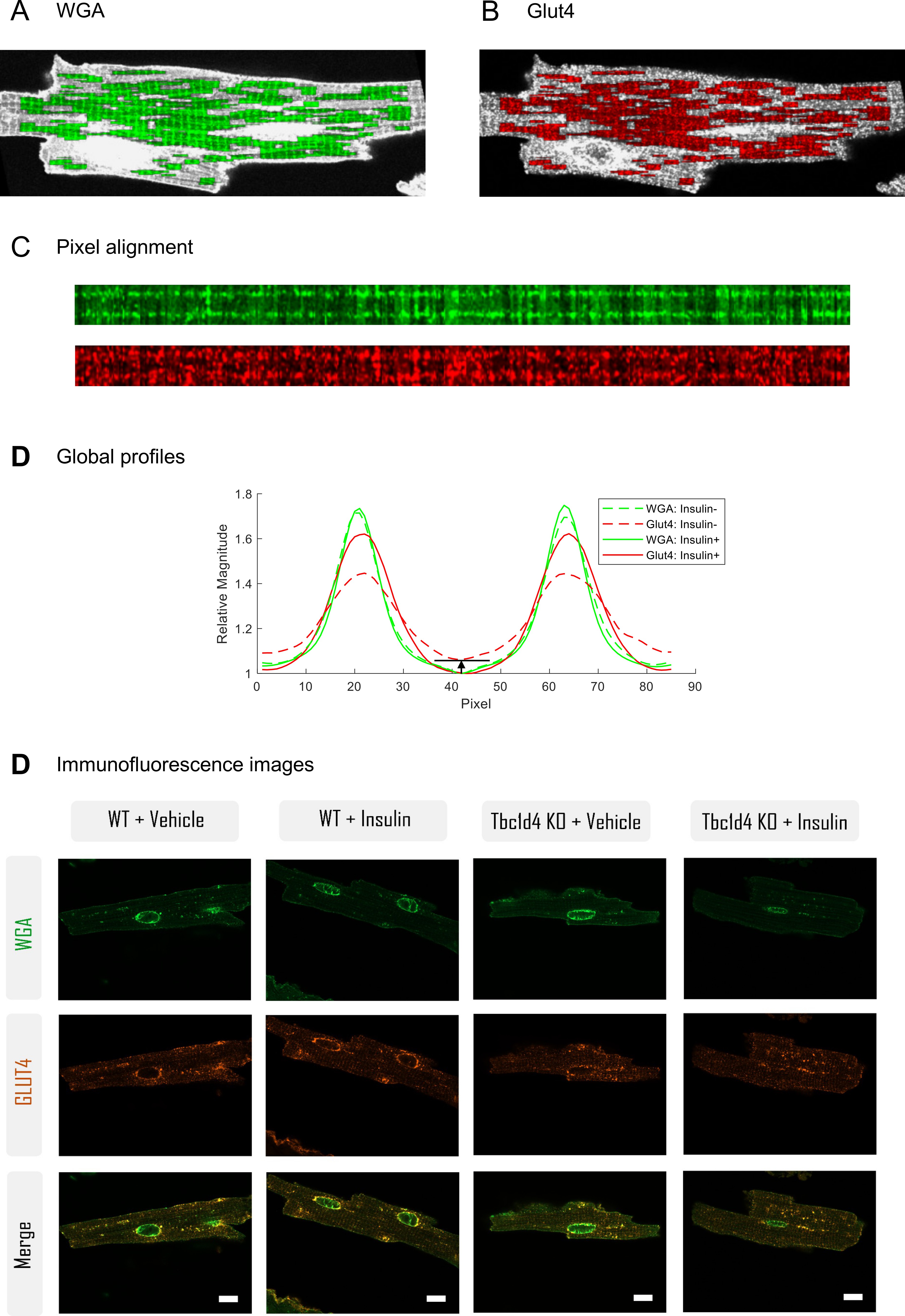

Supplement: Supplementary file 7 — Supplementary Figure S4. Fluorescence imaging-based single-cell co-localization data processing. [file 12933_2024_2338_MOESM7_ESM.jpg]

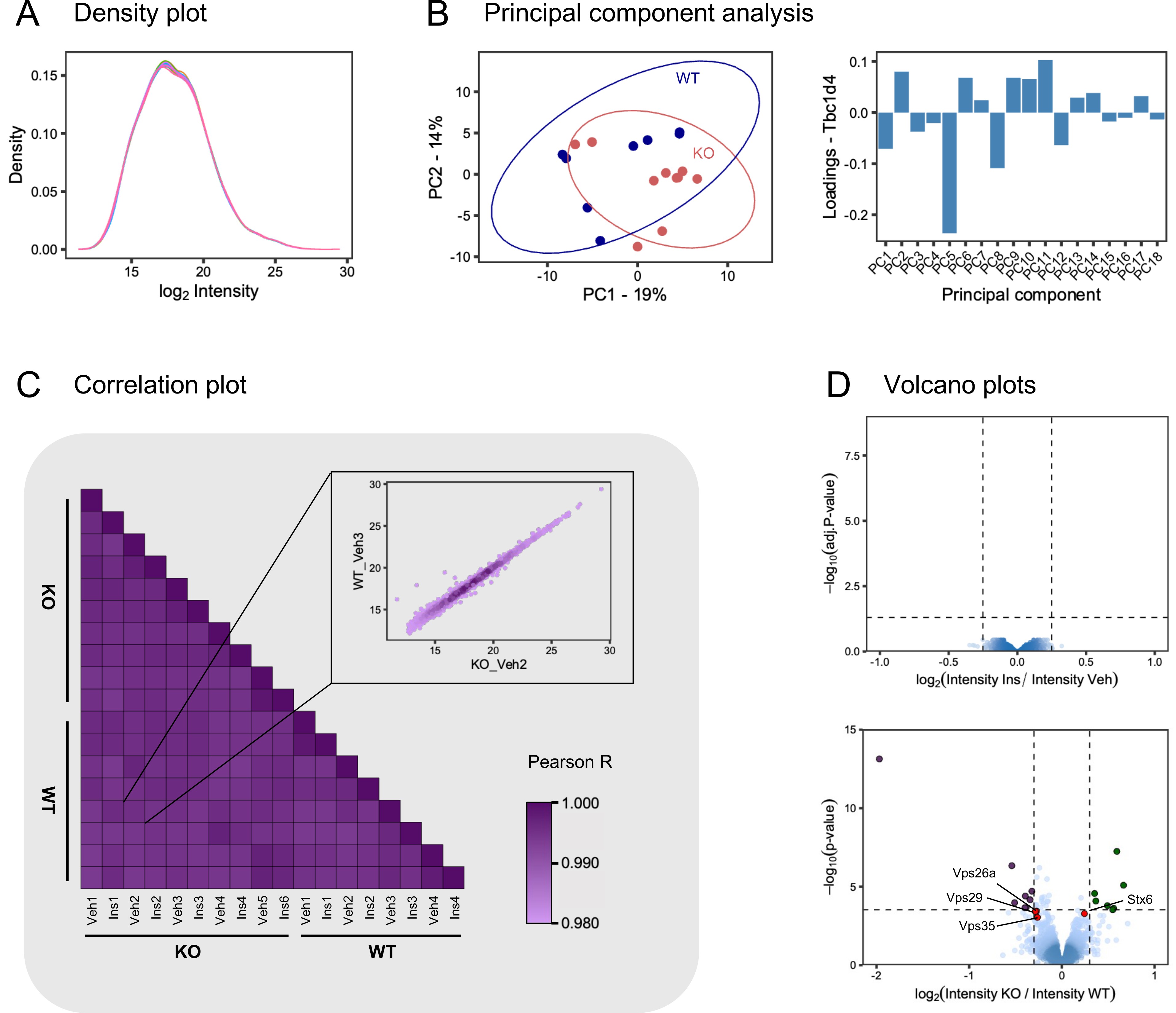

Supplement: Supplementary file 8 — Supplementary Figure S5. Quality control of Tbc1d4 KO and WT cardiomyocyte proteome measurements. [file 12933_2024_2338_MOESM8_ESM.jpg]

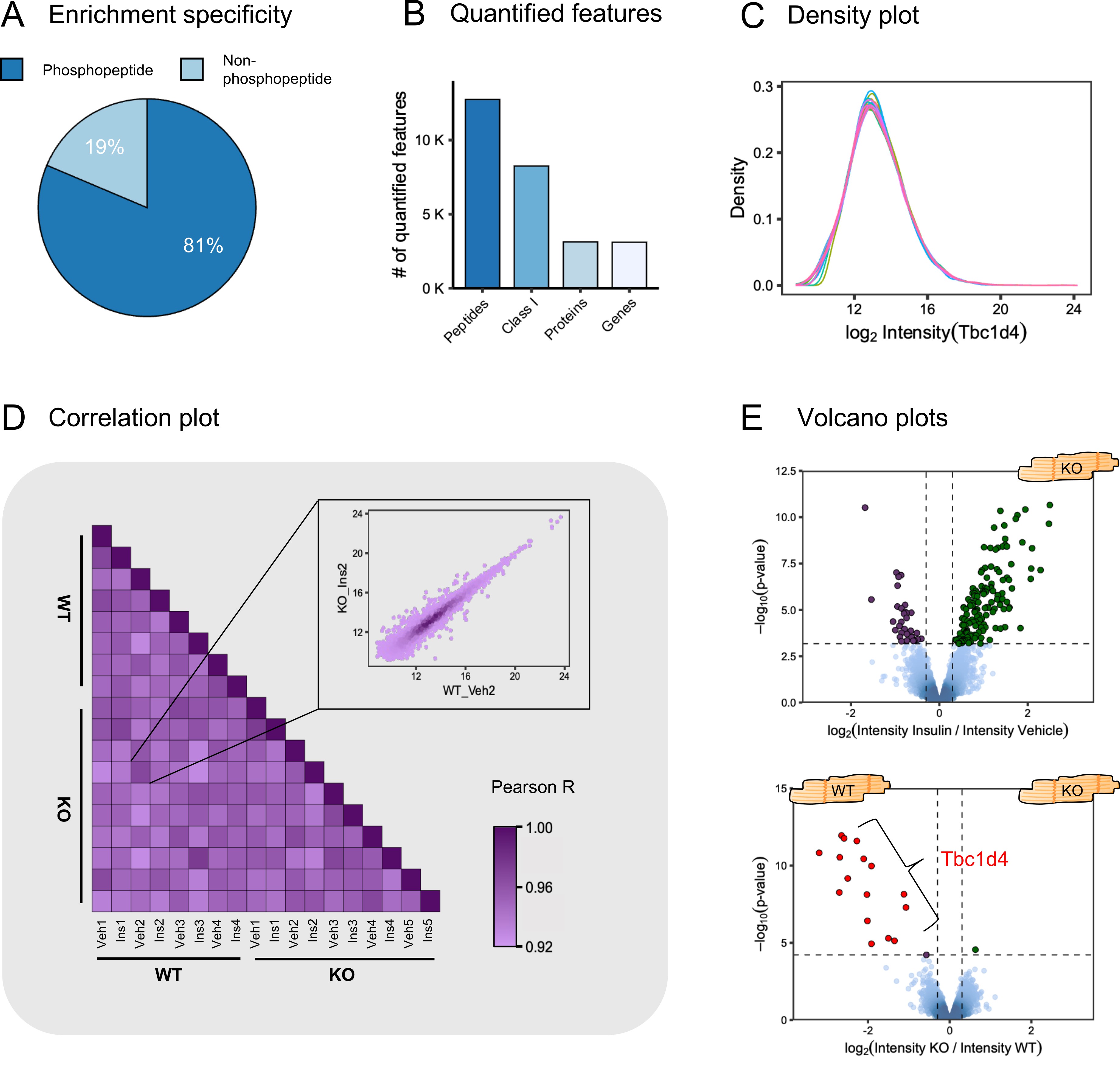

Supplement: Supplementary file 9 — Supplementary Figure S6. Quality control of Tbc1d4 KO and WT cardiomyocyte phosphoproteome measurements. [file 12933_2024_2338_MOESM9_ESM.jpg]
