# Supplementary material for: In-depth phosphoproteomic profiling of the insulin signaling response in heart tissue and cardiomyocytes unveils canonical and specialized regulation
Source: Cardiovasc Diabetol. 2024 Jul 18;23:258. doi: 10.1186/s12933-024-02338-4 (PMC11264841; doi:10.1186/s12933-024-02338-4)
Supplement: Supplementary file 10 — Supplementary Material & Methods. [file 12933_2024_2338_MOESM10_ESM.docx]

**Contents**

[Insulin stimulation of mice and cardiac tissue collection 2](#_Toc160997655)

[Sample preparation for quantitative phosphoproteome measurements of cardiac tissue 2](#_Toc160997656)

[Animals protocol authorization 4](#_Toc160997657)

[Muscle specific Tbc1d4 knock out mice 4](#_Toc160997658)

[Isolation of adult cardiac myocytes 5](#_Toc160997659)

[Culturing of isolated cardiomyocytes and insulin stimulation 5](#_Toc160997660)

[Western blot for validation 6](#_Toc160997661)

[Glut4 and WGA co-staining 6](#_Toc160997662)

[Imaging of Glut4 translocation to Transverse-Tubules 6](#_Toc160997663)

[Phosphoproteome and proteome workflow in isolated cardiac myocytes 10](#_Toc160997664)

[Mass spectrometry data analysis 12](#_Toc160997665)

[Bioinformatics analysis of (phospho)proteomics data 12](#_Toc160997666)

[Data availability 14](#_Toc160997667)

[Supplementary Figures and Legends 14](#_Toc160997668)

[Supplementary Table Legends 16](#_Toc160997669)

[References 16](#_Toc160997670)

# **Insulin stimulation of mice and cardiac tissue collection**

On the day of the experiment, C57Bl6/JRj mice (male, 13 weeks old) were fasted for 4 h (from 9 am until 1pm). At the beginning of the experiment, mice were sedated using isoflurane (induction dose 4%, and maintenance dose 2.5%). When sufficiently sedated (absence of reflexes), mice were opened at the midline of the abdomen and the vena cava was exposed. The mice then received 0.75 IU/Kg body weight of insulin (Humulin® R, U-100, Eli Lilly; IN, USA) or an equal volume of saline by intra venous injection through the vena cava. Insulin dosage was determined in accordance with established standard dosages used in mice insulin tolerance tests to estimate insulin action in vivo[1-3]. After 10 minutes, the heart (left and right ventricle) was collected, washed in cold phosphate-buffered saline and snapfrozen in liquid nitrogen. After tissue collection, the mice were scarified by cervical dislocation.

Blood glucose was measured with a handheld glucose meter (Accu-Chek®) from the tail vein after anaesthesia induction in the chamber and then again at the tissue collection time-point (10 minutes after insulin exposure). Group differences were assessed before and after injection. Data are expressed as mean ± standard deviation (SD). Mann-Whitney-test was used to compare independent samples (significance level .05). Prism v.9.4.1 (SAS Institute, Abacus Concept Inc.; CA, USA) was used to generate graphs and calculate statistics.

# **Sample preparation for quantitative phosphoproteome measurements of cardiac tissue**

Tissue collection and protein extraction

Left and right ventricles were collected from hearts obtained from mice treated with saline (n=5) and mice treated with insulin (n=6). Sample preparation was carried out as described earlier**[4]**. Briefly, tissue samples were homogenized in lysis buffer (50 mM Tris HCl pH 8.5, 5 mM EDTA, 150 mM NaCl, 10 mM KCl, 1% Triton-X 100, 5 mM NaF, 5 mM beta-glycerophosphate, 1mM Na-orthovanadate, 1x Complete Protease Inhibitor cocktail (Roche; CH) using a combination of 2.8 mm and 1.4 mm zirconium oxide beads on a pre-cooled Precellys homogenizer (Bertin Technologies; FR) in reinforced 2ml tubes. The homogenates were incubated at 4°C for 2 h with rotation at 20 rpm and subsequently spun at 10,000xg for 10 min at 4°C to clear the lysate. The protein amounts were determined using Pierce BCA Protein Assay Kit (Thermo Fisher Scientific; MA, USA). Protein precipitation was carried out by adding ice-cold acetone (4:1, v/v), incubated for 1h at -20°C and spun at 2,000xg for 2 min. The pellet was washed once with ice-cold acetone and resuspended in GndHCl buffer (6M GndHCl in 50 mM Tris pH 8.5) by intermittent vortexing, sonicating and heating at 95°C. Samples were reduced and alkylated using 5 mM TCEP and 10 mM CAA for 15 min in dark at room temperature (RT).

Peptide preparation

Samples were diluted to a final concentration of 2M GndHCl using 50 mM Tris pH 8.5 and pre-digested with Lysyl Endopeptidase® C (Fujifilm Wako Chemicals Corp; JP) at 1:50 enzyme:protein ratio and incubated for 2 h at RT, 750 rpm. Samples were again diluted to a final concentration of 0.5M GndHCl using 50 mM Tris pH 8.5 and digested using trypsin (Life technologies; CA, USA) at 1:100 enzyme:protein ratio for 16 h at 37 °C, 750 rpm. The reaction was quenched by addition of trifluoroacetic acid (TFA) to a final of concentration of 0.5% and samples were centrifuged at high speed (14,000 xg, 10 min) to sediment debris. Peptides were desalted and concentrated on C18 SepPak columns (Waters Corp.; MA, USA). Peptides were eluted twice with 40% and 60% acetonitrile (ACN), and organic solvents were subsequently evaporated by vacuum centrifugation (Eppendorf). Peptides were resuspended in 50 mM HEPES pH8.5. Peptide concentration was estimated using Pierce BCA Protein Assay Kit.

TMT labelling

TMT labelling was carried out following a published protocol**[5].** Briefly, 100 µg of peptides from each sample were diluted to equal final volumes with MQ water. Each 20 µl peptide sample was mixed with 200 µg TMT 11-plex (Thermo Fisher Scientific) dissolved in 100% anhydrous ACN. The reaction was carried out at 3.33 µg/µl peptide, 20% ACN, and pH 8.0 for 1h at 1,000 rpm, RT. Each reaction was quenched using a final concentration of 1% hydroxylamine and incubated at RT for 15 min at 1,000 rpm. The 11 samples were pooled, for the following phosphopeptide enrichment.

Phosphopeptide enrichment

Phosphopeptide enrichment was carried out as previously described**[6],** with the following slight modifications. The volume of the pooled peptide sample was adjusted to 1ml with enrichment buffer (20 mg/ml 2,5-dihydroxybenzoic acid, 80% ACN, 6% TFA) and spun at 16,000 xg for 15 min to remove any debris. TiO_2_ beads (titanium dioxide beads, 5 μm Titansphere, GL Sciences; JP) were preincubated in enrichment buffer (1 mg of beads/10 µl of enrichment solution) for 20 min at RT, added to the peptide mix (1:4, sample:bead ratio w/w), and incubated for 15 min while rotating. After incubation, the slurry was centrifuged at 1,000 xg for 5 min, and the supernatant was collected in a separate tube. The beads were then washed with 800µl wash buffers A-E (wash buffer A: 80% ACN and 6% TFA; wash buffer B: 50% ACN and 6% TFA; wash buffer C: 80% ACN and 1% TFA; wash buffer D: 50% ACN and 1% TFA; wash buffer E: 10% ACN and 1% TFA), one after the other, spun at 1,000 xg for 5 min. Supernatants were collected and subjected to second incubation with fresh bead slurry preincubated and washed as described above. Beads from each enrichment were loaded on pre-conditioned C8 STAGE tip (3M Empore; MN, USA). The phosphorylated peptides were eluted with 30μl of 15% NH_4_OH followed by 30 μl of 15% NH_4_OH/40% ACN. Enrichment 1 and 2 were then combined and vacuum dried to a volume below 10µl.

Off-line peptide fractionation

The enriched peptide sample was fractionated by off-line peptide fractionation. One volume of 50mM ammonium bicarbonate (ABC) was added to the sample. Then fractionation was carried out by micro-flow reverse-phase ultra-performance liquid chromatograpy (UPLC) on an Dionex UltiMate 3000 UPLC system (Thermo Fisher Scientific) equipped with an Dionex Ultimate 3000 using a Thermo Acclaim PA2 column (3 µm, 3.0 mm x 150 mm) using a flow rate of 30 µl/min. A linear gradient was employed consisting of buffer A (5 mM ammonium bicarbonate (VWR; PA, USA), pH8) and B (100% ACN, VWR): 0-62 min: 8-28% B, 62-67 min: 28-60% B, 67-70 min: 60-70% B isocratic, 70-78 min: 70-8% B, followed by column re-equilibration at 8% B for 9 minutes. Outflow from 1-70 minutes was collected in 1-minute intervals into 12 concatenated fractions in the autosampler. The fractions were acidified with 10 μl 5% formic acid (FA) and solvent was removed by vacuum centrifugation. Fractions were resuspended in 2% ACN/0.1 % TFA.

## **Mass spectrometry measurements of cardiac tissue phosphoproteome samples**

##

Samples were analysed by online reversed-phase liquid chromatography coupled to an Orbitrap Exploris™ 480 mass spectrometer (LC-MS/MS, Thermo Fisher Scientific). Approximately 200 ng of each fraction was autosampled using a nanoflow Easy-nLC system (Proxeon Biosystems). Peptides were delivered to an in-house pulled and packed 50 cm fused-silica emitter column with reversed-phase ReproSil-Pur C18-AQ 1.9μm resin (Dr. Maisch GmbH). Peptides were separated for 60 min using a multi-step linear gradient (Buffer A: 0.1% formic acid, Buffer B: 0.1% formic acid in ACN; 5-25% Buffer B in 45 min, 25-40% Buffer B in 5 min, 40-80% Buffer B in 5 min, 80% Buffer B 5 min). Column effluent was directly ionized in a nano-electrospray ionization source operated in positive ionization mode and electrosprayed into the mass spectrometer. Spray voltage was set to 2 kV, funnel RF level at 40, and heated capillary temperature at 275 °C. Full-MS spectra (350-1400 m/z) were acquired at 60,000 MS resolution and full MS AGC target was 300% with a maximum fill time of 25 ms. A data-dependent Top20 method then sequentially isolated the most intense precursor ions for higher-energy collisional dissociation (HCD) in an octopole collision cell. MS/MS spectra of fragment ions were subsequently recorded at 30,000 resolution and AGC target 200% in the Orbitrap (maximum fill time of 54 ms) with normalized collision energy set to 30%. Minimum intensity threshold was kept at 2E5, charge state 2-6 and isolation width was set at 0.7 m/z.

# **Animals protocol authorization**

All experiments were approved by the Ethics Committee of the Ministry of Food, Agriculture and Fisheries of Denmark (license number 2020-15-0201-00508, 2019-15-0201-01659 and 2018-15-0201-01397) and local Ethics Committee at the University of Copenhagen (local project plan P22-574 for cardiomyocyte isolation, P20-361 for in vivo insulin stimulation, A23-252 breeding project plant, and P22-254 for tissue harvest).

# **Muscle specific Tbc1d4 knock out mice**

*Tbc1d4* gene is predominantly expressed in two isoforms (short and long). The short isoform is lacking exon 11 and 12. The long isoform is primarily expressed in skeletal and heart muscle[7, 8]. Long isoform-specific *Tbc1d4*-KO mice were generated by introducing a frameshift mutation in exon 11 using CRISPR-Cas9 technology as previously described[9]. **Briefly, F1.129S2;C57BL6/N embryonic stem cells were transfected with gRNA (AGAAGGATGTGTGAAGGCTTGG) and donor template. ESC clones carrying the mutation were identified by PCR using the following primer seq-TBC1D4-FW: TCGCAGCCCTTCAAGGAAAG and Seq-TBC1D4-RV: TCACTCTGAAATTAACCGGCCA**. After chimeric mice generation, an IVF of C57Bl6/N oocytes was carried out with sperm from fertile males deriving from clone #69. Chimeric male founders in hybrid F1.129S2;C57BL/6N genetic background were bred to C57BL/6NRj females to generate F1 pups. Next generations were produced by sibling inter-crossing so the resulting colony was established in a mixed background with a major component of C57BL/6NRj (75%). Tbc1d4 KO cohorts were generated crossing heterozygous animals. The knockout mouse model is genotyped using the following primer pair: Seq-FW: TCGCAGCCCTTCAAGGAAAG & Seq-13d-RV: GCAGAGAAGGATGTGTGCTGC. Immunoblot analyses demonstrate loss of TBC1D4 protein in heart and skeletal muscle but not in adipose tissue from the long isoform-specific Tbc1d4-KO mouse model[9].

# **Isolation of adult cardiac myocytes**

The studies in isolated cardiomyocytes were designed such that we could investigate the signaling effect of insulin, but also the effect of Tbc1d4 KO. Accordingly, cardiomyocytes were isolated from Tbc1d4 KO animals as well as from their wild type littermates. This design was chosen to ensure the same genetic background in both Tbc1d4 KO and wildtype groups. For the cardiomyocyte isolations, mice were injected with heparin (Leo Pharma) 500 IU i.p and were anesthetized with 150 mg/kg pentobarbital. Five minutes later, when sufficiently sedated (absence of reflexes), hearts were collected and kept on ice-cold perfusion buffer[10] (150mM NaCl, 14.7mM KCl, 0.6mM KH_2_PO_4_, 0.6mM Na_2_HPO_4_, 1.2 mM MgSO_4_-7H_2_O, 10mM Na-HEPES, 4.6mM NaHCO_3_, 30mM Taurine, 10mM BDM and 5.5mM glucose, all from Sigma) containing heparin (500 U heparin to 50 ml perfusion buffer) and 40 µM Ca^2+^. The heart was cleaned by gravity by ice-cold perfusion buffer containing heparin after the aorta was cannulated with an aortic mouse cannula (Harvard apparatus 73-2816-OD 1.0mm, Harvard apparatus) and tied with a double-knot using silk 6-0 silk thread. Once cannulated and connected with the perfusion system, retrograde perfusion with calcium free buffer was performed for approximately 4 min at 37 C (flow rate of 4mL/min). After 4 min, the perfusion with collagenase type 2 (Wortington Biochemical) at 1.8 mg/ml and containing 40 µM CaCl_2_ at 37°C was started. After 6 min of exposure to collagenase, the ventricles were separated from atria and collected in a 6 cm dish and teased apart in 10-12 pieces in stopping buffer (perfusion buffer supplemented with 10% calf serum and 12.5µM CaCl_2_). Cells were collected in a 15 ml tube and diluted up to 10 ml with stopping buffer. To separate cardiac myocytes from non-myocytes, cells were centrifuged for 3 minutes at 20 g.

# **Culturing of isolated cardiomyocytes and insulin stimulation**

After 15 min of calcium reintroduction (400µM CaCl_2_ in stopping buffer followed by 900µM CaCl_2_ in stopping buffer), isolated cardiomyocytes from WT or *Tbc1d4* KO mice were resuspended in plating medium (10% calf serum, 10mM BDM [Sigma], 100U/ml penicillin, MEM [Gibco, 2157-022]). Cells were plated on round 1.5 coverslips (VWR) at the bottom of a 6-well plate treated with 10 µg/ml of laminin (Corning) (for staining) or 60mm dishes (for phosphoproteome sample preparation) coated with 10 µg/ml laminin (Corning). After 1h at 37°C 5%CO_2_, the media was exchanged to culture medium (0.1% BSA (Sigma), 100U/ml penicillin, 2 mM Glutamine in MEM). Cardiomyocytes from each isolation were separated and treated with vehicle (ddH_2_O) and 10 nM bovine insulin (Sigma) for 30 min in the cell incubator[11].

# **Western blot for validation**

Protein lysates were homogenized as previously described. Proteins were quantified using Pierce Rapid Gold BCA kit. 50 µg protein per lane was separated on sodium dodecyl sulfate polyacrylamide electrophoresis (SDS-PAGE) 4-12% Bis-Tris gels with MOPS buffer (Invitrogen). Proteins were wet-transferred to a 0.45 µm PVDF-FL membrane (Millipore) for 1h at 30V using a transfer buffer 10% methanol. Membranes were blocked 1h at RT with Odyssey PBS blocking buffer (Licor Biosciences) and incubated ON at 4ºC with the following primary antibodies against: GSK a/b (Cell Signaling Technology [CST], 5676), phospho GSKa/b S21/9 (CST, 8566), vinculin (R&D systems, MAB6896), RPSKB1 (CST, 34475), phospho RPSKB1 T412 (CST, 9205), phospho AKT S473 (CST, 4060) and AKT (CST, 4691). Immunodetection was performed with secondaries goat anti-rabbit or mouse IgG conjugated with IRDyes 680RD or 800CW (Licor Biosciences) (dilution 1:5000 or 1:10,000 respectively).

Images were captured using a CLX Odyssey Imaging system version 5.2.5 (Licor Biosciences). The resolution was 169 µm and the focus offset was 0.0 mm. Images were analysed using Image Studio Lite software version 5.2.5. The background was local, determined as the median of intensities at the top and bottom of the box. Data are presented as mean ± SD. Two-tailed Student´s t-test was used to compare independent samples (significance level .05). Prism v.9.4.1 (SAS Institute, Abacus Concept Inc., Berkeley, CA, USA) was used to generate graphs and calculate statistics. Normality was tested with Shapiro-Wilk test and Kolmogorov-Smirnov test (significance level .05)

# **Glut4 and WGA co-staining**

After insulin (or saline) treatment, cells were washed once with PBS (Ca, Mg free, pH 7.4) and fixed with 4% paraformaldehyde pH 7.4 (Sigma) for 15 min at RT. Cell membrane was stained for 10 min RT with Wheat Germ agglutinin (WGA) conjugated with Alexa 488 (Invitrogen), dilution 1:50. Cells were washed with PBS three times and permeabilized for 10min RT with PBS 0.2% Triton X100 (Sigma). Cells were blocked with blocking buffer (10% normal donkey serum in PBS) 1h RT and incubated ON 4ºC with Rabbit polyclonal anti-GLUT4 (Invitrogen, PA5-23052), dilution 1:500. Cells were washed with PBS and incubated 1h RT with secondary antibody anti-Rabbit Alexa Fluor 568 (Abcam, 175470) dilution 1:400. After washing, coverslips were mounted with Prolong diamond (Invitrogen).

# **Imaging of Glut4 translocation to Transverse-Tubules**

Confocal laser scanning microscopy imaging of WGA and Glut4 immuno-staining was performed with a Zeiss LSM980 microscope fitted with an Airyscan2 detector (Zeiss, Oberkochen, Germany), using a Plan-Apochromat 63x/1.4 NA oil immersion objective, and 488 and 561 nm solid-state lasers. Pixel dwell time was 0.86 µs/px, sampling was set to Nyquist (43 nm/px in XY, 150 nm in Z), and 9 planes were acquired as Z stacks. Fluorescent signals were collected by the aligned Airyscan detector, using 573-620 and 499-577 nm spectral range for Alexa Fluor 568 and 488 respectively. All images were acquired at the same magnification and pixel resolution for all cells at 0.0425 micron/pixel.

The raw Airyscan images were 3D-reconstructed using the algorithm built-in into Zeiss’ ZEN Blue 3 software. The strength of the Wiener filter was estimated by reconstructing selected images and set to 8 for both Alexa Fluor 488 and Alexa Fluor 568 channels.

## **Quantification of Glut4 co-localization with t-tubules**

In cardiomyocytes, Glut4 preferentially translocates to the t-tubules in response to insulin stimulus[12]. To assess this, we quantified Glut4 co-localization with the t-tubules through analysis of confocal images of fluorescence-intensity from cardiomyocytes co-stained for the membrane (WGA, conjugated to a green fluorescent dye) and Glut4 (conjugated to a red fluorescent dye). Image pixels corresponding to membrane transverse-tubules were identified by the following: Cell images were rotated such that the long-axis of the cell was aligned to the horizontal resulting in t-tubules being vertically aligned. As WGA staining of the t-tubules was locally punctate, a smoothing kernel was applied in the vertical direction (aligned with t-tubules) to augment t-tubule appearance over background fluorescence. A template was constructed of fluorescence intensity changes over the horizontal direction corresponding to 2 t-tubules (and immediate proximity) when the cardiomyocyte is fixed at rest under the image magnification and pixel resolution used here. The same template was used to detect t-tubules for all cells. This template was used in a cross-correlation kernel along the image rows, reaching a local peak when best aligned to 2 t-tubules in the t-tubule-augmented image. Local cross-correlation peaks with a minimum correlation coefficient of 0.8 were taken to signify a local pixel row-segment overlying 2 t-tubules. As the correlation coefficient ignores the absolute range of intensity values, this included some false positive detections where intensity fluctuated in the same direction as the template but to a minimal extent, e.g. in the nuclei. These false positives were identified and removed as follows: The cross-correlation-identified pixel row-segments were aligned and the distribution of fluorescence intensity at the peaks and the central troughs was assessed. As the range of intensity values in the troughs was relatively narrow (within the same log10 decade), the peak values were normalized by their respective trough values, and this distribution was assessed for median and median absolute deviation. Pixel row-segments where the trough-normalized peak intensity was between -1*1.483*MAD to +3*1.483*MAD about the median were retained. This quality control removed the majority of false positive detections from the cross-correlation kernel. After this culling, the final set of aligned pixel row-segments corresponding to 2 t-tubules from the augmented membrane staining image was used to collect the same pixel row-segments from the corresponding original (non-augmented) WGA and Glut4-staining images. At each pixel location across the aligned row-segments, the 75^th^ percentile of fluorescence intensity was taken to construct the representative fluorescent signals for both WGA and Glut4 staining; 1 each per cell.

These signals were then prepared for quantifying both peak magnitude and concordance of Glut4 to WGA at the t-tubules as follows: The raw 75^th^ percentile signals were normalized by the central trough value so that peak magnitude is relative to background intensity thereby mitigating any minimal cell-to-cell differences in staining, illumination, or other handling. The resultant signals had their DC component (=1) removed. After this, the WGA signal alone was normalized by its vector-magnitude. From these signals, the Glut4-magnitude correlation with WGA at the t-tubules was calculated as:

$$Glut4/WGA co-localization metric =\frac{Glut4\bullet\mathrm{WGA}}{\left| \mathrm{WGA} \right|}$$

Glut4 may be proximal to the t-tubules prior to translocation. This proximity coupled with optical convolution from the imaging system would produce an un-stimulated Glut4 profile that is already well-correlated to the WGA profile. Upon stimulation, it would be expected that Glut4 translocation to the t-tubule would marginally enhance this correlation, but furthermore, would also increase the relative magnitude of Glut4 in the peak regions at the expense of the shoulder and trough regions (constant integral under the curve pre- and post-translocation). The metric above is sensitive to and would increase due to either effect.

## **Statistical analysis**

The metric above is sensitive to re-organization of Glut4, however, the absolute value of the metric would also be dependent on relative Glut4 expression. Glut4 expression is presumably relatively consistent amongst ventricular cardiomyocytes from the same animal, but could have greater variability between animals and even more so between genotypes. Consequently, we chose a statistical evaluation that is sensitive to the relative changes in the above metric between vehicle- versus insulin-treated cells from the same animal while ignoring the specific values of the metric from one animal to the next. Specifically, we chose the Mann-Whitney U-test of data ranks between two groups and applied this on a per-animal basis. The standard Mann-Whitney U-test disregards direction, i.e. if one group contains the smaller data values and one group contains the larger, it produces a small test statistic regardless of which group contains the larger data values. However, we wanted to assess for a consistent increase in the metric with insulin treatment over vehicle treatment across animal groups. As such we calculated the one-sided Mann-Whitney U-statistic and the associated specific probability (and cumulative) density function (PDF/CDF) of this statistic for each of the coupled vehicle- versus insulin-treated groups (i.e. per animal). From these functions we determined the total probability (i.e. relative frequency) with which the lower-ranked data values would separate into the vehicle-treated group and the higher-ranked data values into the insulin-treated group to the same (or more extreme) extent by chance alone if all data values had been drawn from the same distribution; i.e. P(U≤u) under the null hypothesis. This probability was calculated for each of the coupled vehicle- versus insulin-treated groups (i.e. per animal). We then assessed the compound cumulative probability per-genotype, e.g. for wildtype (WT):

Compound-P_WT_ = P_WT1_ * P_WT2_ * … * P_WTn_

and the corollary for the tbc1d4-knockout group (KO). We also calculated the associated specific CDF for the compound probability for each genotype: As the cumulative probabilities from a Mann-Whitney style U-statistic of ranks has a dis-continuous PDF that is non-zero at a finite number of discrete points and is specific to the numbers of observations in each of the two groups (vehicle vs. insulin treated), we numerically calculated the integrals to find the CDF for the product of cumulative-probabilities as random variables arising from independent U-statistic distributions. Specifically, if X and Y are the cumulative probabilities arising from two distributions, e.g. two distinct U-distributions U_A_ and U_B_:

$$X=P\left( U_{A}\leq u \right) and Y=P(U_{B}\leq u)$$

then X and Y are themselves distributed over [0 1] such that, specifically for X:

$$PDF: f_{X}\left( x \right)=P\left( X=x \right)=\left\{ \begin{aligned} 0, &x<0 \\ 0, &x>1 \end{aligned} \right.$$

$$CDF: F_{X}\left( x \right)=P\left( X\leq x \right)=\left\{ \begin{aligned} 0, &x<0 \\ 1, &x>1 \end{aligned} \right.$$

and similarly for Y. As such, the CDF of the product of these two variables (Z=X*Y):

$$CDF: F_{Z}\left( z \right)=P\left( Z\leq z \right)=P\left( XY\leq z \right)=\int_{-\infty}^{0} f_{X}\left( x \right)\int_{z/x}^{\infty} f_{Y}\left( y \right) dy dx+\int_{0}^{\infty} f_{X}\left( x \right)\int_{-\infty}^{z/x} f_{Y}\left( y \right) dy dx$$

reduces to:

$$F_{Z}\left( z \right)=\int_{0}^{x=z} f_{X}\left( x \right) dx+\int_{x=z}^{1} f_{X}\left( x \right) F_{Y}\left( \frac{z}{x} \right) dx$$

which for the case where X and Y are distributed discretely over [0 1] such that:

$$f_{X}\left( 0\leq x\leq1 \right) \left\{ \begin{aligned} \neq0, &x=[q_{1}, q_{2}, \ldots, q_{i}, \ldots q_{N}] \\ =0, otherwise \end{aligned} \right.$$

$$F_{X}\left( 0\leq x\leq1 \right) increases in a staircase-like manner at x=[q_{1}, q_{2}, \ldots, q_{i}, \ldots q_{N}]$$

$$F_{Y}\left( 0\leq\frac{z}{x}\leq1 \right) increases in a staircase-like manner at \frac{z}{x}=[p_{1}, p_{2}, \ldots,p_{k},\ldots p_{M}]$$

then the above reduces further to:

$$F_{Z}\left( z \right)=F_{X}\left( z^{-} \right) +\sum_{\forall q_{*}\geq z \left\{ \begin{aligned} q_{i} \ldots q_{N} if z=q_{i} \\ q_{i+1} \ldots q_{N} if q_{i}>z>q_{i+1} \end{aligned} \right.} f_{X}\left( q_{*} \right)F_{Y}\left( \frac{z}{q_{*}} \right)$$

where F_X_(z^–^) indicates that the lower-limit value should be used at the discontinuity when z=q_i_. F_Z_(z) only needs to be calculated at all z=q_i_ and all z=q_i_*p_k_ to be fully described.

This numerical integration process was iterated until all *n* distributions were incorporated, i.e.:

$$F_{U_{1}*U_{2}}\left( z \right)=\int_{0}^{x=z} \begin{aligned} f_{U_{2}}\left( x \right) dx+ \int_{x=z}^{1} f_{U_{2}}\left( x \right) F_{U_{1}}\left( \frac{z}{x} \right) dx \\ \end{aligned}$$

$$F_{U_{1}*U_{2}*U_{3}}\left( z \right)=\int_{0}^{x=z} \begin{aligned} f_{U_{3}}\left( x \right) dx+ \int_{x=z}^{1} f_{U_{3}}\left( x \right) F_{U_{1}*U_{2}}\left( \frac{z}{x} \right) dx \\ \end{aligned}$$

etc. (where U_j_ indicates the cumulative-probability as random variable arising from the distinct, independent, one-sided U-distribution associated with the specific coupled vehicle- versus insulin-treated group ‘*j*’) until F_U1*U2*…*Un_(z) was known. From this final compound CDF, we calculated the total probability with which the data values would consistently separate into the vehicle and treated groups as observed (or more extremely) by chance alone if within each of the coupled vehicle- versus insulin-treated groups (i.e. per animal) all data values had been drawn from the same distribution. As it would be notable if the insulin-treated metric was consistently *less* than the vehicle across groups to a similar extent, we calculated the genotype p-val based on twice the compound-probability, i.e.:

Genotype p-val = P( Z ≤ 2 * P_1_ * P_2_ * … * P_n_ ) under the null hypothesis.

# **Phosphoproteome and proteome workflow in isolated cardiac myocytes**

Cardiomyocytes from WT (n=4) and Tbc1d4 KO (n=5) mice were isolated, and each preparation was divided in two equal portions. One portion was treated with vehicle, while the other received insulin for 30 min, following the procedure described above. After incubation, cardiomyocytes were washed twice with PBS and collected in 200 µl boiling lysis buffer (6M Gnd-HCl, 5 mM TCEP, 10 mM CAA, 100 mM Tris-HCl pH 8.5). The samples were heated at 99ºC for 10 min and sonicated (15 cycles of 15s on/off, Bioruptor homogenizer, Diagenode). Protein concentration was determined by BCA assay and protein amounts were equalized across all samples. The proteins were predigested with Lys-C (1:50 w/w) for a period of 2h at 25ºC, 700 rpm, after dilution to 2M Gnd-HCl. Subsequently, enzymatic digestion with trypsin (1:100 w/w) was carried out overnight at 37ºC, 700 rpm, at a final concentration of 0.5M GndHCl. Protease activity was quenched by adjusting the pH to ~1 with 10% TFA.

Peptides were desalted on pre-conditioned Sep-Pak C18 cartridges (Waters), eluted with 40% ACN, followed by 60% ACN, and dried by vacuum centrifugation. Peptides were resuspended in 50 mM HEPES, pH 8 prior to TMT labelling as described above (TMTpro 18-plex, final reaction: 4µg/µl peptides, 20% ACN). After labelling, samples were quenched with 1% hydroxylamine for 15 min, RT. Samples were pooled, and 80 µg peptides were desalted on a Sep-Pak C18 cartridge before fractionation for proteome analysis. Phosphopeptide enrichment was performed as previously described with the remaining pooled sample. Briefly, peptides were diluted 4-fold in enrichment buffer (20 mg/ml DHB, 80% ACN, 6% TFA) and TiO_2_ beads (GL Sciences) were activated for 20 min in enrichment buffer. Peptides were mixed with beads at a bead:peptide ration of 4:1 for 15 min, RT. Beads were repeatedly washed, phosphopeptides were eluted and a second enrichment was performed with the unbound peptides following the same procedure.

Unenriched and enriched peptide samples were fractionated using an EASY-nLC 1000 (Thermo Fisher Scientific) equipped with a 2.6 µm EVO C18 100 Å Kinetex reversed phase column (150 x 0.3mm) (Phenomenex). For phosphoproteome measurements, 8 concatenated fractions were collected, whereas 24 fractions were obtained for global proteome measurements. Approximately 20 µg of peptides were separated at a flow rate of 2 µl/min with a gradient starting from 3% B, which was linearly increased to 40% B within 57 min, 60% B within 5 min, 95% B within 10 min, held at 95% B for 10 min, decreased to 3% B in 10 min and kept at 3% B for 8 min. Fraction collection and concatenation occurred automatically at 30-second intervals in a 96-well plate, and was achieved by coupling the column outlet to an Opentrons OT-2 pipetting robot (Opentrons Labworks), controlled by an in-house python script. Buffer A: 10 mM TEAB, Buffer B: 80% ACN, 10 mM TEAB. Peptide fractions were dried by vacuum centrifugation and reconstituted in 2% ACN and 0.1% TFA.

## **Mass spectrometry measurements**

LC separation was performed using a Vanquish Neo UHPLC system (Thermo Fisher Scientific). Peptides were loaded on a 25 cm x 75 µm Aurora Gen2 column (IonOpticks) packed with C18 resin (1.6 µm) and separated using a 101-min gradient at a flow rate of 400 nL/min. The gradient was ramped to 5% B within two minutes, followed by a linear increase to first 17% B over 55 min and then 25% B in 21 min. The percentage of B was further ramped up to 35% over 13 min before reaching 85% B in 3 min, concluding with a final column wash for 7 min. The column temperature was maintained at 50ºC. Peptides eluting from the column were ionized via electrospray ionization in a nano-electrospray ionization source, operated in positive ionization mode, and introduced into an Orbitrap Ascend^TM^ Tribrid^TM^ mass spectrometer (Thermo Fisher Scientific) equipped with the FAIMS Pro Duo^TM^ Interface. Positive ion spray voltage was set to 2.6 kv and the FAIMS CV was fixed at -45 for all acquisitions with a 4.6 L/min carrier gas flow and default charge state set to 2. The ion transfer tube temperature was maintained at 275ºC. MS1 scans (400-1600 m/z) were acquired in the Orbitrap at 60,000 resolution, with a maximum injection time of 123 ms and a normalized AGC target of 100%. Monoisotopic peak determination (MIPS) was performed in peptide mode and only peaks with an intensity >2.5e4 and charge state 2-6 were selected for MS2. MS2 scans (110-2000 m/z) were acquired at a resolution of 45,000, with a maximum injection time of 91 ms and the normalized AGC target set to 200%. Dynamic exclusion was enabled with an exclusion duration of 60 s, mass tolerance of 10 ppm and “exclude isotopes” option. The cycle time was limited to 2.4 s. Normalized HCD collision energy was 35% and precursor ions were isolated with a 0.7 m/z quadrupole window.

# **Mass spectrometry data analysis**

Raw MS data were converted to mzML format using the ProteoWizard MSConvert tool v3.0.23006[13]. Files were searched with FragPipe (v20.0/21.1) using the built-in “TMT16” and “TMT16-phospho” workflows for isolated cardiomyocyte proteome and phosphoproteome measurements, respectively. FragPipe’s “TMT10-phospho” workflow was employed for bulk tissue phosphoproteome measurements. MSFragger (v3.8/4.0)[14] was used to search the files against a mouse fasta database containing all reviewed SwissProt protein entries and appended with common contaminants as well as an equal number of decoys (Uniprot ID: UP000000589). Phosphorylation of serine/threonine/tyrosine residues and methionine oxidation were set as a variable modification, TMT labeling of the N-terminus and lysine as well as carbamidomethylation of cysteine were treated as fixed modifications. A maximum of three variable modifications per peptide was allowed, and trypsin was selected as the protease with up to two missed cleavages. The minimum peptide length was set to seven amino acids, peptide and fragment mass tolerance to 20 ppm. Peptide-spectrum matches (PSMs) were validated with Percolator[15] and PTM sites were localized with PTMProphet[16]. All outputs were filtered to 1% false discovery rate (FDR) in Philosopher (v5.0.0/5.1.0)[17]. TMT report ion intensities were extracted and summarized with TMT-Integrator. The label type was adjusted to TMT-18 and TMT-11 depending on the experiment. For ratio-to-abundance conversion, “Use MS1 intensity” and “Top 3 ions” options were disabled, and the minimum purity threshold was increased to 0.75. The log2-transformed and normalized intensities used for downstream processing in R were obtained through “global normalization” within TMT Integrator, which involves median centering and scaling by median absolute deviation.

# **Bioinformatics analysis of (phospho)proteomics data**

## Differential expression analysis

Statistical processing of quantitative proteomics data was conducted using R version 4.3.2. Differential abundance analysis was performed using the R package LIMMA v3.58.1[18], employing an empirical Bayes moderated t-test statistics. For experiments involving comparison within and between subjects (treatment + genotype), the correlation between repeated observations was estimated using the duplicateCorrelation function and incoporated into the linear model. Features were deemed regulated if their intensity comparisons between groups had an FDR-adjusted p-value < 0.05 (Benjamini-Hochberg correction) for the isolated cardiomyocyte experiments and an adjusted p-value < 0.1 for the bulk phosphoproteomic experiments. In addition, an absolute log_2_ fold-change cut-off greater than 0.3 was used.

## Functional enrichment analysis

Functional enrichment analysis was performed using the R clusterProfiler package v4.10.0[19]. Briefly, over-representation analysis of gene ontology biological processes (GOBP) was conducted on the unique protein identifiers of differentially expressed features using hypergeometric testing against an experiment-specific background. Enrichment results were filtered for terms with BH corrected p-values < 0.05 and were then used to calculate pairwise term similarities based on Jaccard’s similarity. Functional grouping was achieved using the treeplot function in the R package enrichplot v1.22.0. Here, hierarchical clustering was based on Ward’s distance. The name of each cluster was assigned based on the most significantly enriched term within itself.

## Kinase-substrate enrichment analysis

A comprehensive mouse kinase substrate database was assembled by integrating data from the following resources using OmnipathR v3.10.1[20]: PhosphoSitePlus[21], KEA[22], phosphoELM[23], and SIGNOR[24]. Interactions without literature references and kinases comprising less than 4 substrates were removed. Additionally, kinases not listed in the “Uniprot kinase list” (<https://www.uniprot.org/docs/pkinfam.txt>) were excluded from the database. The processed phosphopeptide tables were subsequently filtered to retain only entries with localized phosphorylation sites, and peptides were collapsed to single-site format. Kinase activities were inferred using fast gene set enrichment analysis (fgsea) implemented in the R decoupleR package v2.8.0[25]. A minimum number of 4 substrates per kinase was required and significance was claimed at a 5% FDR.

## Intersection with single-cell RNA sequencing data

To understand the cellular origins of proteins that exhibit significantly increased phosphorylation upon insulin stimulation, we utilized the Heart Cell Atlas v2, a largescale dataset comprising single-cell RNA sequencing (scRNA-seq) data from more than 700k cells and nuclei from 25 adult donors[26]. We queried this dataset for genes corresponding to proteins with significantly increased phosphorylation following insulin stimulation. For this, we mapped the mouse gene names to their human orthologs using a combination of the Ensembl biomart mapping and converting the gene name to uppercase letters[27]. Expression across cell types was visualized as a dot plot using Python v3.10 and Scanpy v1.9.1[28]. Additionally, we computed the average expression levels of each gene per cell type and specifically visualized the expression profiles of INSR and TBC1D4 using bar charts.

## Network representation

Proteins with upregulated phosphosites were represented as a STRING network using Cytoscape v3.10.1[29] and the plugins StringApp v2.0.2[30] and Omics Visualizer v1.3.1[31], and subsequently polished in Adobe Illustrator 2024. Briefly, phosphopeptides with at least one localized phosphorylation site were ranked by t-test statistic and the top150 entries were selected for visual representation.

Curation of a canonical insulin signaling gene set

A comprehensive gene set comprising proteins involved in canonical insulin signaling was curated through integration of knowledge from four resources: Gene Ontology Biological Processes (GOBP), Kyoto Encyclopedia of Genes and Genomes (KEGG), Reactome, and WikiPathways (WP). Utilizing OmnipathR v3.10.1, annotations were retrieved and gene sets filtered to include those specifically associated with insulin signaling pathways. The final list encompassed 488 unique proteins and was used to classify proteins into canonical and non-canonical insulin signaling pathway.

# **Data availability**

The mass spectrometry proteomics data have been deposited to the ProteomeXchange Consortium via the PRIDE[32] partner repository with the dataset identifier PXD050545.

# **Supplementary Figures and Legends**

## **Supplementary Figure 1. Quality control for bulk phosphoproteomics data**

**(A)** Specificity of phosphopeptide enrichment. Pie chart illustrating the proportion of peptides identified with phosphorylation modifications (phosphopeptides) and non-phosphorylated peptides relative to the total number of identified peptides (N = 14,975). **(B)** Number of quantified features with observed phosphorylation events. Peptides: Number of phosphopeptides class I: Subset of phosphorylated peptides with a site localization probability ≥ 0.75, Proteins: Number of proteins covered by all phosphopeptides, Genes: Number of genes represented by the phosphopeptides. **(C)** Distribution of intensities for all quantified phosphopeptides across individual samples. Intensities have been log2-transformed and normalized prior to visualization. **(D)** Principal component analysis (PCA) plot for the dataset. Plotted are the coordinates of all samples along the first three PCs. Left: Color-coding indicates the treatment variable (Control vs Insulin). Right: Color-coding by the log2-intensity of the phosphopeptide Insr_Y1175Y1179Y1180.

## **Supplementary Figure 2. Functional enrichment analysis.**

Network analysis of gene ontology biological processes (GO-BP) activated upon insulin stimulation. Terms are represented as nodes, with edges denoting Jaccard’s similarity index. Aggregation of related terms into 5 distinct clusters was achieved via hierarchical clustering of the pairwise term similarities, using Ward’s method. Each cluster is named after the most significantly enriched GO-BP term within it. The relative cluster size was represented as a bar chart as shown in main figure 2.

## **Supplementary Figure 3. Proteomic and phosphoproteomic analysis of Tbc1d4-deficiency.**

**(A)** Schematic workflow for sample preparation. From left to right: Primary cardiac myocytes from control (N=4) and Tbc1d4-knockout (N=5) mice were isolated. Half the cells were stimulated with insulin (30 min), while the other half received saline, resulting in a total of 9 insulin-treated and 9 control samples. Proteins were extracted and enzymatically cleaved into peptides, which were multiplexed with tandem mass tags (TMTpro). A fraction of these peptides was fractionated and analyzed by LC-MS for proteome measurements. The remaining peptides were enriched for phosphopeptides prior to fractionation and LC-MS. **(B)** Validation of significantly regulated targets in adult murine cardiomyocytes. Representative blots showing p-GSK3α^S21^/p-GSK3β^S9^ (left panel) and p-AKT^S473^ (right panel) from freshly isolated WT cardiomyocytes treated with vehicle or 10 nM insulin for 30 min. Quantification of p-GSK/total GSK ratio for each isoform or p-AKT/total AKT ratio. Data represents mean ± SD. Independent t-test *** p < 0.0002; **** p < 0.0001.

## **Supplementary Figure 4. Fluorescence imaging-based single-cell co-localization data processing.**

**(A)** Confocal fluorescence-intensity imaging slice of an insulin-treated cardiomyocyte immune-stained against wheat germ agglutinin (WGA) with a green fluorescent dye to highlight cell membrane structures. A computer algorithm detects constant-width pixel row-segments centered over staining of two consecutive t-tubules (false-colored in green). **(B)** Fluorescence intensity image of immuno-staining against Glut4 with a red fluorescent dye from the same cell slice. The same pixels as highlighted in (A) are false-colored in red. **(C)** The aligned pixel row-segments highlighted in (A) and (B) - only a small subset is shown here and rotated 90 degrees for display purposes. **(D)** Using the pixel alignment, pixels are combined along the vertical direction in (C) to create a global characteristic profile of WGA (membrane; green) and Glut4 (red) staining across two consecutive t-tubules [corresponding to combining pixels along the horizontal direction in (C) to produce a vertical line-scan]. Profiles are normalized by the trough fluorescence level in the trough between the two t-tubule aligned peaks. With insulin treatment (solid line traces), Glut4-fluorescence has a profile that closely matches t-tubule WGA staining. Without insulin treatment (dashed line traces), the trough-level-normalized Glut4 fluorescence profile typically has a lesser trough-to-peak amplitude. For the no-insulin Glut4 profile to have the same total area under the curve as the insulin-treated Glut4 profile (representing the same total amount of Glut4-fluorescence), the profile would need to be offset from 1 (black arrow). As such, the no-insulin cells have a more even spatial distribution of Glut4 compared to the insulin-treated cells where Glut4 has re-distributed to be more aligned with the t-tubules. WGA traces have been magnitude scaled for display. **(E)** Glut4 staining in WT and Tbc1d4 deficient cardiomyocytes. Representative images of freshly isolated cardiomyocytes treated with insulin or vehicle co-stained for Glut4 and WGA. WGA staining (row 1, green); Glut4 staining (row 2, red); merged channels (row 3). Scale bar = 10 µm.

**Supplementary Figure 5. Quality control of Tbc1d4 KO and WT cardiomyocyte proteome measurements.**

Mass spectrometry-based proteome data was acquired from cardiomyocytes isolated from WT or Tbc1d4 KO animals. The cardiomyocytes were either stimulated with insulin or vehicle prior to LC-MS measurements. In total there were 18 samples multiplexed by TMTpro labels. Refer to Supplementary Figure S3 for experimental details. **(A)** Density plot of log-transformed protein intensities measured for all samples. **(B)** Principal component analysis. Left: Plot of the first two PCs. Right: Loadings of Tbc1d4 across all 18 PCs. **(C)** Scatter plots and pairwise Pearson correlation coefficients across samples. **(D)** Volcano plot of differentially expressed proteins. Horizontal lines indicate an FDR-adjusted p-value ≤ 0.05, vertical lines indicate absolute log2FC > 0.3. Top: Volcano plot of proteins differentially expressed proteins in response to insulin stimulation. No protein passes the significant cut-off. Bottom: Volcano plot of proteins differentially expressed in response to knockout of Tbc1d4. Selected proteins are highlighted in red.

**Supplementary Figure 6. Quality control of Tbc1d4 KO and WT cardiomyocyte phosphoproteome measurements.**

Mass spectrometry-based phosphoproteome data was acquired from cardiomyocytes isolated from WT or Tbc1d4 KO animals. The cardiomyocytes were either stimulated with insulin or vehicle prior to LCMS measurements. In total there were 18 samples multiplexed by TMTpro labels. Refer to Supplementary Figure S4 for experimental details. **(A)** Specificity of phosphopeptide enrichment. Pie chart illustrating the proportion of peptides identified with phosphorylation modifications (phosphopeptides) and non-phosphorylated peptides relative to the total number of identified peptides (N = 13,127). **(B)** Number of quantified features with observed phosphorylation events. Peptides: Number of phosphopeptides, class I: Subset of phosphorylated peptides with a site localization probability ≥ 0.75, Proteins: Number of proteins covered by all phosphopeptides, Genes: Number of genes represented by the phosphopeptides. **(C)** Distribution of intensities for all quantified phosphopeptides across individual samples. Intensities have been log2-transformed and normalized prior to visualization. **(D)** Heatmap of pairwise Pearson correlation coefficients across samples as well as exemplary scatter plot. **(E)** Volcano plot of differentially expressed phosphopeptides. Horizontal lines indicate an FDR-adjusted p-value ≤ 0.05, vertical lines indicate absolute log2FC ≥ 0.3. Top: Volcano plot of phosphorylated peptide differentially expressed in response to insulin stimulation in Tbc1d4-knockout (KO) cardiomyocytes. Significantly up-regulated phosphopeptide are highlighted in green, down-regulated phosphopeptides in purple. Bottom: Volcano plot showing phosphopeptides with significant differences between KO and WT cardiomyocytes. Highlighted in red are differentially abundant peptides mapping to Tbc1d4.

**Supplementary Table Legends**

Supplementary Table S1: List of phosphopeptides quantified upon acute insulin stimulation in murine cardiac tissue. Results of functional enrichment analyses.

Supplementary Table S2: List of proteins identified and quantified in isolated cardiomyocytes.

Supplementary Table S3: List of phosphopeptides quantified upon insulin stimulation in isolated cardiomyocytes. Results of functional enrichment analyses.

# **References**

1. McGuinness OP, Ayala JE, Laughlin MR, Wasserman DH: **NIH experiment in centralized mouse phenotyping: the Vanderbilt experience and recommendations for evaluating glucose homeostasis in the mouse**. *Am J Physiol Endocrinol Metab* 2009, **297**(4):E849-855.

2. Li N, Guenancia C, Rigal E, Hachet O, Chollet P, Desmoulins L, Leloup C, Rochette L, Vergely C: **Short-term moderate diet restriction in adulthood can reverse oxidative, cardiovascular and metabolic alterations induced by postnatal overfeeding in mice**. *Sci Rep* 2016, **6**:30817.

3. Mathews ST, Singh GP, Ranalletta M, Cintron VJ, Qiang X, Goustin AS, Jen KL, Charron MJ, Jahnen-Dechent W, Grunberger G: **Improved insulin sensitivity and resistance to weight gain in mice null for the Ahsg gene**. *Diabetes* 2002, **51**(8):2450-2458.

4. Linscheid N, Logantha S, Poulsen PC, Zhang S, Schrolkamp M, Egerod KL, Thompson JJ, Kitmitto A, Galli G, Humphries MJ *et al*: **Quantitative proteomics and single-nucleus transcriptomics of the sinus node elucidates the foundation of cardiac pacemaking**. *Nat Commun* 2019, **10**(1):2889.

5. Zecha J, Satpathy S, Kanashova T, Avanessian SC, Kane MH, Clauser KR, Mertins P, Carr SA, Kuster B: **TMT Labeling for the Masses: A Robust and Cost-efficient, In-solution Labeling Approach**. *Mol Cell Proteomics* 2019, **18**(7):1468-1478.

6. Lundby A, Andersen MN, Steffensen AB, Horn H, Kelstrup CD, Francavilla C, Jensen LJ, Schmitt N, Thomsen MB, Olsen JV: **In vivo phosphoproteomics analysis reveals the cardiac targets of beta-adrenergic receptor signaling**. *Sci Signal* 2013, **6**(278):rs11.

7. Baus D, Heermeier K, De Hoop M, Metz-Weidmann C, Gassenhuber J, Dittrich W, Welte S, Tennagels N: **Identification of a novel AS160 splice variant that regulates GLUT4 translocation and glucose-uptake in rat muscle cells**. *Cell Signal* 2008, **20**(12):2237-2246.

8. Moltke I, Grarup N, Jorgensen ME, Bjerregaard P, Treebak JT, Fumagalli M, Korneliussen TS, Andersen MA, Nielsen TS, Krarup NT *et al*: **A common Greenlandic TBC1D4 variant confers muscle insulin resistance and type 2 diabetes**. *Nature* 2014, **512**(7513):190-193.

9. Kjobsted R, Chadt A, Jorgensen NO, Kido K, Larsen JK, de Wendt C, Al-Hasani H, Wojtaszewski JFP: **TBC1D4 Is Necessary for Enhancing Muscle Insulin Sensitivity in Response to AICAR and Contraction**. *Diabetes* 2019, **68**(9):1756-1766.

10. O'Connell TD, Rodrigo MC, Simpson PC: **Isolation and culture of adult mouse cardiac myocytes**. *Methods Mol Biol* 2007, **357**:271-296.

11. Graveleau C, Zaha VG, Mohajer A, Banerjee RR, Dudley-Rucker N, Steppan CM, Rajala MW, Scherer PE, Ahima RS, Lazar MA, Abel ED: **Mouse and human resistins impair glucose transport in primary mouse cardiomyocytes, and oligomerization is required for this biological action**. *J Biol Chem* 2005, **280**(36):31679-31685.

12. Davey KA, Garlick PB, Warley A, Southworth R: **Immunogold labeling study of the distribution of GLUT-1 and GLUT-4 in cardiac tissue following stimulation by insulin or ischemia**. *Am J Physiol Heart Circ Physiol* 2007, **292**(4):H2009-2019.

13. Adusumilli R, Mallick P: **Data Conversion with ProteoWizard msConvert**. *Methods Mol Biol* 2017, **1550**:339-368.

14. Kong AT, Leprevost FV, Avtonomov DM, Mellacheruvu D, Nesvizhskii AI: **MSFragger: ultrafast and comprehensive peptide identification in mass spectrometry-based proteomics**. *Nat Methods* 2017, **14**(5):513-520.

15. Käll L, Canterbury JD, Weston J, Noble WS, MacCoss MJ: **Semi-supervised learning for peptide identification from shotgun proteomics datasets**. *Nat Methods* 2007, **4**(11):923-925.

16. Geiszler DJ, Kong AT, Avtonomov DM, Yu F, Leprevost FDV, Nesvizhskii AI: **PTM-Shepherd: Analysis and Summarization of Post-Translational and Chemical Modifications From Open Search Results**. *Mol Cell Proteomics* 2021, **20**:100018.

17. da Veiga Leprevost F, Haynes SE, Avtonomov DM, Chang HY, Shanmugam AK, Mellacheruvu D, Kong AT, Nesvizhskii AI: **Philosopher: a versatile toolkit for shotgun proteomics data analysis**. *Nat Methods* 2020, **17**(9):869-870.

18. Kammers K, Cole RN, Tiengwe C, Ruczinski I: **Detecting Significant Changes in Protein Abundance**. *EuPA Open Proteom* 2015, **7**:11-19.

19. Wu T, Hu E, Xu S, Chen M, Guo P, Dai Z, Feng T, Zhou L, Tang W, Zhan L *et al*: **clusterProfiler 4.0: A universal enrichment tool for interpreting omics data**. *Innovation (Camb)* 2021, **2**(3):100141.

20. Türei D, Korcsmáros T, Saez-Rodriguez J: **OmniPath: guidelines and gateway for literature-curated signaling pathway resources**. *Nat Methods* 2016, **13**(12):966-967.

21. Hornbeck PV, Zhang B, Murray B, Kornhauser JM, Latham V, Skrzypek E: **PhosphoSitePlus, 2014: mutations, PTMs and recalibrations**. *Nucleic Acids Res* 2015, **43**(Database issue):D512-520.

22. Lachmann A, Ma'ayan A: **KEA: kinase enrichment analysis**. *Bioinformatics* 2009, **25**(5):684-686.

23. Dinkel H, Chica C, Via A, Gould CM, Jensen LJ, Gibson TJ, Diella F: **Phospho.ELM: a database of phosphorylation sites--update 2011**. *Nucleic Acids Res* 2011, **39**(Database issue):D261-267.

24. Perfetto L, Briganti L, Calderone A, Cerquone Perpetuini A, Iannuccelli M, Langone F, Licata L, Marinkovic M, Mattioni A, Pavlidou T *et al*: **SIGNOR: a database of causal relationships between biological entities**. *Nucleic Acids Res* 2016, **44**(D1):D548-554.

25. Badia IMP, Vélez Santiago J, Braunger J, Geiss C, Dimitrov D, Müller-Dott S, Taus P, Dugourd A, Holland CH, Ramirez Flores RO, Saez-Rodriguez J: **decoupleR: ensemble of computational methods to infer biological activities from omics data**. *Bioinform Adv* 2022, **2**(1):vbac016.

26. Kanemaru K, Cranley J, Muraro D, Miranda AMA, Ho SY, Wilbrey-Clark A, Patrick Pett J, Polanski K, Richardson L, Litvinukova M *et al*: **Spatially resolved multiomics of human cardiac niches**. *Nature* 2023, **619**(7971):801-810.

27. Martin FJ, Amode MR, Aneja A, Austine-Orimoloye O, Azov Andrey G, Barnes I, Becker A, Bennett R, Berry A, Bhai J *et al*: **Ensembl 2023**. *Nucleic Acids Research* 2022, **51**(D1):D933-D941.

28. Wolf FA, Angerer P, Theis FJ: **SCANPY: large-scale single-cell gene expression data analysis**. *Genome Biology* 2018, **19**(1):15.

29. Shannon P, Markiel A, Ozier O, Baliga NS, Wang JT, Ramage D, Amin N, Schwikowski B, Ideker T: **Cytoscape: a software environment for integrated models of biomolecular interaction networks**. *Genome Res* 2003, **13**(11):2498-2504.

30. Doncheva NT, Morris JH, Gorodkin J, Jensen LJ: **Cytoscape StringApp: Network Analysis and Visualization of Proteomics Data**. *J Proteome Res* 2019, **18**(2):623-632.

31. Legeay M, Doncheva NT, Morris JH, Jensen LJ: **Visualize omics data on networks with Omics Visualizer, a Cytoscape App**. *F1000Res* 2020, **9**:157.

32. Perez-Riverol Y, Bai J, Bandla C, García-Seisdedos D, Hewapathirana S, Kamatchinathan S, Kundu DJ, Prakash A, Frericks-Zipper A, Eisenacher M *et al*: **The PRIDE database resources in 2022: a hub for mass spectrometry-based proteomics evidences**. *Nucleic Acids Res* 2022, **50**(D1):D543-d552.
